# DNA methylation patterns at birth predict health outcomes in young adults born very low birthweight

**DOI:** 10.1186/s13148-023-01463-3

**Published:** 2023-03-23

**Authors:** Vicky A. Cameron, Gregory T. Jones, L. John Horwood, Anna P. Pilbrow, Julia Martin, Chris Frampton, Wendy T. Ip, Richard W. Troughton, Charlotte Greer, Jun Yang, Michael J. Epton, Sarah L. Harris, Brian A. Darlow

**Affiliations:** 1 Christchurch Heart Institute, Department of Medicine, University of Otago, Christchurch, PO Box 4345, Christchurch, 8140 New Zealand; 2 Department of Surgical Sciences, University of Otago, Dunedin, New Zealand; 3 Christchurch Health and Development Study, Department of Psychological Medicine, University of Otago, Christchurch, New Zealand; 4 Department of Paediatrics, University of Otago, Christchurch, New Zealand; 5 Respiratory Physiology Laboratory, Christchurch Hospital, Christchurch, New Zealand

**Keywords:** DNA methylation, Epigenetics, Birthweight, Cardiovascular, Risk prediction

## Abstract

**Background:**

Individuals born very low birthweight (VLBW) are at increased risk of impaired cardiovascular and respiratory function in adulthood. To identify markers to predict future risk for VLBW individuals, we analyzed DNA methylation at birth and at 28 years in the New Zealand (NZ) VLBW cohort (all infants born < 1500 g in NZ in 1986) compared with age-matched, normal birthweight controls. Associations between neonatal methylation and cardiac structure and function (echocardiography), vascular function and respiratory outcomes at age 28 years were documented.

**Results:**

Genomic DNA from archived newborn heel-prick blood (*n* = 109 VLBW, 51 controls) and from peripheral blood at ~ 28 years (*n* = 215 VLBW, 96 controls) was analyzed on Illumina Infinium MethylationEPIC 850 K arrays. Following quality assurance and normalization, methylation levels were compared between VLBW cases and controls at both ages by linear regression, with genome-wide significance set to *p* < 0.05 adjusted for false discovery rate (FDR, Benjamini-Hochberg). In neonates, methylation at over 16,400 CpG methylation sites differed between VLBW cases and controls and the canonical pathway most enriched for these CpGs was Cardiac Hypertrophy Signaling (*p* = 3.44E^−11^). The top 20 CpGs that differed most between VLBW cases and controls featured clusters in *ARID3A*, *SPATA33*, and *PLCH1* and these 3 genes, along with *MCF2L*, *TRBJ2*-1 and *SRC*, led the list of 15,000 differentially methylated regions (DMRs) reaching FDR-adj significance. Fifteen of the 20 top CpGs in the neonate EWAS showed associations between methylation at birth and adult cardiovascular traits (particularly LnRHI). In 28-year-old adults, twelve CpGs differed between VLBW cases and controls at FDR-adjusted significance, including hypermethylation in *EBF4* (four CpGs), *CFI* and *UNC119B* and hypomethylation at three CpGs in *HIF3A* and one in *KCNQ1*. DNA methylation GrimAge scores at 28 years were significantly greater in VLBW cases versus controls and weakly associated with cardiovascular traits. Four CpGs were identified where methylation differed between VLBW cases and controls in both neonates and adults, three reversing directions with age (two CpGs in *EBF4,* one in *SNAI1* were hypomethylated in neonates, hypermethylated in adults). Of these, cg16426670 in *EBF4* at birth showed associations with several cardiovascular traits in adults.

**Conclusions:**

These findings suggest that methylation patterns in VLBW neonates may be informative about future adult cardiovascular and respiratory outcomes and have value in guiding early preventative care to improve adult health.

**Supplementary Information:**

The online version contains supplementary material available at 10.1186/s13148-023-01463-3.

## Background

Very low birthweight (VLBW, < 1500 g) babies account for nearly 2% of all births [1], with a large proportion of VLBW survivors remaining healthy as adults. However, being born VLBW or very preterm is a chronic condition, such that VLBW individuals are at risk of cardiovascular and metabolic disorders becoming manifest at an earlier age than their term-born peers [2–6]. Data from very large Scandinavian population registries show that preterm birth (< 37 weeks’ gestation), particularly very low gestational age (< 32 weeks), is associated with increased mortality in young adulthood [2, 6, 7], with respiratory, endocrine, and cardiovascular disorders among the main causes of death [2]. Recent publications of Mendelian randomization [8] and genome-wide association studies [9] have shown that lower birthweight is causally related to increased risk of cardiovascular disease and type-2 diabetes (DM2). Many studies, including meta-analyses, in VLBW or very premature cohorts have reported elevated blood pressures in young adulthood [10, 11]. Premature birth has been associated with risk of adult pulmonary hypertension with an odds ratio of 3.08 after adjusting for known risk factors [3, 12]. Moreover, VLBW and preterm individuals may have altered cardiac structure and function that persists into young adulthood and beyond. The Victorian Infant Collaborative Study (VICS) reported that former extremely preterm (< 28-week gestation) adolescents aged 18 years had decreased left ventricular (LV) mass and cavity size but preserved cardiac function [13]. Cardiac magnetic resonance imaging (MRI) studies have reported that relative to term-born peers, young adults (20–39 years) born preterm had increased LV mass but smaller LV cavities, associated with impaired LV systolic/diastolic function [14, 15], and smaller right ventricles (RV), with increased RV wall mass, resulting in reduced RV ejection fraction [16]. The mechanism leading to smaller heart chambers may be a consequence of the preterm birth prematurely halting cardiac maturation, with early termination of muscle cell proliferation within the myocardium [17], resulting in a reduction in the absolute number of cardiomyocytes and reduced functional reserve. Low birthweight is reportedly also associated with impaired endothelial function in young adults [18, 19], with greater right carotid intima-media thickness [20]. In short, cardiovascular features documented in the VLBW population consistently include elevated systolic blood pressure, smaller heart dimensions and impaired cardiac and vascular function. Crucially, in a registry-based study of over 2.6 million individuals born in Sweden between 1987 and 2012 [21] and other studies [22], preterm birth was linked to a substantial increase in the risk of early heart failure. Compared to those born at term (≥ 37-week gestation), adjusted relative risks for heart failure incidence were RR = 17.0 after extremely preterm birth (< 28 weeks) and 3.58 after very preterm birth (28–31 weeks) [21].

In addition to altered cardiovascular function, those born VLBW or very preterm have enduring abnormal respiratory function as young adults, with reduced expiratory airflow and an increased risk of future obstructive airways disease [23, 24]. The developmental origins of lung disease are well-recognized [25] and may interact with and compound adverse cardiovascular health outcomes in VLBW adults. Moreover, the impact of a “second hit” on the lung, such as tobacco smoking, may exacerbate both perinatal lung damage [25] and suboptimal cardiovascular health.

We have recently described the cardiovascular and lung function, as well as metabolic characteristics of young adults (mean age 28 years) in the 1986 NZ VLBW cohort born < 1500 g, compared with 100 age-matched controls of normal birthweight [1, 26–29]. The VLBW individuals were shorter and lighter in stature, and consistent with international studies, had higher systolic blood pressure, smaller hearts, reduced stroke volume and endothelial function, as well as higher LV and arterial elastance than their normal birthweight peers [26, 27]. Adult VLBW cases also showed a higher incidence of airflow obstruction and other features of impaired respiratory function compared with controls [29].

Among VLBW survivors, early detection of those most at risk of pulmonary hypertension and heart failure may provide an opportunity for preventative interventions. At present, there are no reliable markers to predict future risk of cardiovascular or respiratory disorders apart from birthweight and gestation. General approaches to prevention include optimizing postnatal nutrition, with preterm infants exclusively fed human milk showing beneficial effects on cardiac size and function in young adulthood compared to those fed formula [30]. As with the general population, cardiovascular health could also be promoted through supporting VLBW adults to adopt a healthy diet and engage in regular physical activity, and living smoke-free would benefit both cardiovascular and respiratory health [31]. In addition, more intensive monitoring and targeted pharmacological approaches for high-risk individuals may improve myocardial development beyond the critical postnatal window. Although the benefits of antihypertensive therapies, inhibitors of cardiac remodeling or of atherosclerotic disease progression in the VLBW population are unknown, in the general population lowering blood pressure by 2 mm Hg can reduce hypertension by 17%, heart attacks by 6%, and stroke by 15% [32]. Endothelial dysfunction is potentially treatable before it leads irreversibly to atherosclerosis and clinical disease [33]. Thus, a greater understanding of cardiovascular risk stratification is important to inform preventive care for adults born VLBW.

The greater risk of chronic disease in later life results from the convergence of numerous clinical and physiological parameters of the infant and mother, along with life-long environmental exposures. However, the group of individuals born VLBW is very heterogeneous, and the literature is unclear as to which clinical parameters should be used to guide ongoing monitoring and support for the health of VLBW survivors into adulthood. For example, the NZ guidelines for Cardiovascular Disease (CVD) Risk Assessment and Management for Primary Care (2017) [34] make no mention of a patient’s birth history as a trigger for regular assessment of these at-risk individuals. Identification of new biomarkers that are surrogate markers, or even causal markers [35] of the cellular/physiological pathways that underlie susceptibility to chronic disease could lead to better identification of at-risk individuals.

There is now extensive evidence that susceptibility to a number of adult chronic diseases may be linked to the fetal and early postnatal environment through epigenetic processes, such as DNA methylation of cytosine-guanine dinucleotides (CpG) within regulatory elements of genes [36, 37]. Perturbations in DNA methylation have been robustly demonstrated for many disorders (e.g. coronary artery disease [35, 38–40]), physical traits (eg obesity [41–43] and type 2 diabetes [44–46]) and environmental factors (e.g. smoking [47]). DNA methylation patterns associated with heart disease have been used to generate DNA methylation risk scores to predict incident cardiovascular disease [38]. Measures of epigenetic age acceleration, such as DNA methylation (DNAm) GrimAge [48], have been associated with late-life chronic disease including ischemic heart disease [49]. Significantly, being born VLBW has been associated with altered patterns of peripheral blood DNA methylation [37, 50–53], with some methylation differences persisting to adolescence [50, 52] or adulthood [53].

These prior studies indicate that DNA methylation patterns associated with VLBW or preterm birth may persist into adulthood, raising the tantalizing possibility that methylation profiling might be useful to predict adult health and to guide preventative care. Here we aimed to identify methylation markers that differed between NZ VLBW cases and age-matched controls, either at birth or at 28 years, and to determine if these are associated with cardiovascular and respiratory health outcomes in the cohort as young adults. We aimed to identify methylation patterns that might ultimately assist in targeting interventions to mitigate adverse health outcomes in adulthood in those born VLBW.

## Methods

The aim of this study was to determine whether methylation differed between NZ VLBW cases and age-matched controls either at birth or at 28 years of age, and to investigate whether methylation at birth is predictive of cardiovascular and respiratory health outcomes in the VLBW cohort as young adults.

### The New Zealand 1986 VLBW cohort

The New Zealand (NZ) 1986 VLBW cohort originally consisted of all 413 VLBW (< 1500 g) live births admitted to a NZ neonatal unit in 1986, who were enrolled in a prospective audit of retinopathy of prematurity (ROP) with data collected on 173 perinatal variables [1]. Of this cohort 338 (82%) survived to discharge home and were followed up at 7–8 years and at 22–23 years. In 2013–16 (26–30 years), 229 participants (71% of survivors) underwent a 2-day, multidisciplinary evaluation in Christchurch, NZ, as per the study protocol previously described [1, 26, 27, 29]. A flow diagram of inclusion and exclusion criteria is given in Additional file 1: Methods 1. In brief, of the VLBW cases who underwent clinical screening, 64 (28%) were < 1000 g at birth, 57 (25%) were < 28 weeks’ gestation, 72 (31%) were small for gestational age, and 129 (56%) had received antenatal steroids. A control group of 100 subjects were also assessed, who were born healthy at full term (≥ 37 weeks) in 1986 in NZ and had not been admitted to an intensive care unit (recruited through peer nomination by cohort member or random sampling from the electoral rolls, seeking balance with respect to sex, ethnicity, and regional distribution). Participants completed a standardized health questionnaire including self-reported morbidities, and height, weight, body mass index (BMI), and waist-hip measures were recorded. Systolic and diastolic blood pressure were measured in the non-dominant arm by a trained health professional with the participant seated, with the third of three readings over a 15-min period recorded. Transthoracic echocardiography was performed using an iE33 ultrasound machine (Philips Life Healthcare, Amsterdam, The Netherlands) for reporting of indices of cardiac size, structure and function [26, 27]. Endothelial function was assessed by peripheral arterial tonometry using an EndoPAT system (Itamar Medical, Caesarea, Israel), measuring reactive hyperemic index (log normal transformed, LnRHI) [27]. Lung function tests were undertaken as previously described [29] at the Respiratory Physiology Laboratory, Christchurch Hospital, which is accredited by the Thoracic Society of Australia and New Zealand. Peripheral blood samples were collected for subsequent biochemistry, white cell counts and DNA extraction, and whole blood or plasma stored at −80 C. The study was approved by the Southern Health & Disability Ethics Committee (URB/12/05/015), with optional consent obtained for DNA analysis.

### DNA extraction and methylation arrays

Genomic DNA was extracted from archived newborn heel-prick blood spots (3 × 3 mm card punches of “Guthrie” cards) for 109 VLBW and 51 controls, provided by The Neonatal Screening Unit of NZ following patient consent. Blood-spot DNA was extracted with a modified Dried Blood Spots protocol using the QIAamp® 96 Blood Kit (Qiagen, Hilden, Germany), optimized for maximum DNA yield (see Additional files 2: Methods). While DNA shows some degradation after long-term storage, this reportedly does not markedly impact DNA methylation arrays [54]. Genomic DNA was also extracted from peripheral blood at 28 years of age for 215 VLBW cases and 96 controls (those consenting for DNA analysis and for whom blood was available). DNA was isolated from 3 mL frozen whole blood using an automated KingFisher® Flex 24 instrument (ThermoFisher Scientific, Waltham, MA, USA) and Machery-Nagel DNA isolation kits (Machery-Nagel, GmbH &Co, Düren, Germany). All DNA samples underwent bisulfite conversion (EZ DNA Methylation Kit, Zymo, Orange, CA, USA) following the manufacturer’s instructions, prior to hybridization and scanning by an accredited provider (GenomNZ™, AgResearch, Invermay, NZ) on Illumina Infinium Human MethylationEPIC 850 K arrays (Illumina Inc, San Diego, CA, USA), covering 850,000 genome-wide, differentially methylated regions. Cases and controls were run together with balanced physical positions across each array.

#### Methylation data analysis

Raw methylation output files underwent quality assurance (QA) filtering, background and batch normalization and Infinium probe type correction within the Illumina GenomeStudio methylation software module as previously described [55]. In brief, Illumina (Genome Studio) normalized beta values were analyzed for differential methylation using the Chip Analysis Methylation pipeline (ChAMP version 2.20). Any sample flagged as having poor intensity in either the red or green fluorescence channel, probes reported to be cross-reactive and non-specific probes were excluded from the analyses [56], leaving 822,741 CpG sites for analysis. Recorded single nucleotide polymorphism (SNP)-variable CpGs were flagged but not excluded. We subsequently scrutinized the individual distribution patterns of the top sites to ensure that differential methylation observations were not being influenced by obvious SNP effects. The normalized data were analyzed by linear regression to compare VLBW cases and controls, adjusting for sex, blood cell composition (Houseman extended method [57, 58]), EPIC array slide, array position, and CpG call rate (linear). Graphical representation of data with Manhattan plots or principal component analyses (PCA) was performed using Qlucore Omics Explorer bioinformatics software (version 3.4, Lund, Sweden). A shortlist of candidate CpGs for further follow up was generated for each age using a significance threshold after adjusting for false discovery rate (FDR-adj-*p*, Benjamini-Hochberg) of < 0.05. Because of the differences in storage time and method of DNA sample preparation for neonatal blood spots versus adult whole blood, and the potential differences in white cell profiles in newborns versus adults, raw methylation data files were not directly compared between ages. Rather, lists of candidate CpGs that differed between VLBW cases and controls were separately generated for each age, and the resultant lists were compared. In addition, the EWAS data were interrogated for differentially methylated regions (DMRs) using the Bioconductor package DMRcate (https://tinyurl.com/y3uex6bj). All CpGs shortlisted as significantly differentially methylated between VLBW and controls were examined using the UCSC Genome Browser (UCSC Genome Institute, University of California, Santa Cruz, CA, USA) to ensure that they were correctly annotated to their gene location. Gene lists were analyzed for pathway associations and gene networks using Ingenuity® Pathway Analysis (Qiagen, Aarhus, Denmark), after inversing positive/negative fold changes to account for inhibitory methylation effects in this gene expression software, with IPA software recognizing 6053 of 16,400 features in the neonate list and 388 of 1000 features in the adult list. Lists of differentially methylated CpG were also examined for localization relative to CpG islands and with transcription binding sites using DNA Methylation Interactive Visualization Database (DNMIVD) [59]. For the 28-year samples DNA methylation age scores, DNAm GrimAge and GrimAge Adjusted Age [48] were calculated by running the data through the algorithm at https://dnamage.genetics.ucla.edu/home. Comparisons of baseline characteristics between VLBW cases and controls were done using ANOVA and Chi-square tests within SPSS Statistical software for Mac, version 27 (IBM Corporation, Armonk, NY, USA). Association analyses between methylation at our top CpGs and adult cardiovascular and respiratory traits that we have previously reported to differ between VLBW and controls as young adults in this cohort [26, 27] were performed by univariate general linear regression models (GLM) with case–control status, sex and ethnicity as covariates, also using SPSS version 27.

## Results

### Characteristics of the VLBW cases and normal birthweight controls

Characteristics of the subset of VLBW cases and normal birthweight controls for whom DNA was available for methylation analysis at 28 years (215 VLBW, 96 controls) are shown in Table 1. The variables presented are those previously reported to differ significantly between VLBW and controls in the full NZ 1986 VLBW cohort [26, 27, 29]. Mean birthweights of the VLBW cases were approximately a third that of controls. The average gestation at birth in VLBW cases was 29.2 weeks, while all controls self-reported that they were born at term (> 37 weeks, taken as a mean of 38.5 weeks). There was no significant difference in the gender split between VLBW cases and controls, but the distribution of ethnicities differed, with a greater percent of Māori among VLBW cases and a greater proportion of Pacific Peoples and Europeans among controls. At 28 years of age, the VLBW young adults had similar rates of smoking to controls, and similar rates of diabetes and BMI profiles. Among cardiovascular variables at 28 years, VLBW cases had higher systolic BP and MAP, smaller hearts (even when indexed to body surface area, BSA) including: LV mass index (LVMI), right ventricular basal diameter (RV basal dia) and right atrial volume index (RAVI). The VLBW cases also had lower cardiac output (CO), lower stroke volume indexed to BSA (SVI), and lower RV global endocardial longitudinal strain (Endo-GLS), with higher LV elastance and arterial elastance. Table 1 also shows respiratory variables at 28 years, with VLBW having lower z-scores for forced expiratory volume in the first second (FEV1z), forced expiratory flow at 25 and 75% of the pulmonary volume (FEF_25–75_) and FEV1z by FVCz (Tiffeneau-Pinelli index), higher residual volume (RVz) and RVz to total lung capacity (RVz by TLCz), lower diffusing capacity of the lungs for carbon monoxide (DLCOz), lower carbon monoxide transfer coefficient (KCOz), but cases and controls had similar values of VO_2_ Max. Because our study analyzed methylation levels of DNA extracted from peripheral blood mononuclear cells (PBMC), Table 1 also shows that white blood cell profiles were comparable between VLBW cases and controls except for higher neutrophil counts in VLBW young adults.

**Table 1:** Characteristics of VLBW Cases and Controls with DNA Available at 28 years

	VLBW	*n*	Controls	*N*	*p* value
*At birth*					
Birthweight, g	1135 ± 16.1	214	3468 ± 59.9	82	** < 0.001**
Female n (%)	120 (55.8%)	214	61 (63.5%)	96	0.219
Gestation at birth, weeks	29.22 ± 0.17	215	38.50 ± 0 *	96	** < 0.001**
Ethnicity		214		96	**0.003**
Māori	60 (27.9%)		15 (15.6%)		
Pacific	4 (1.9%)		8 (8.3%)		
European	150 (69.8%)		73 (76.0%)		
Ventilation, days	10.69 ± 1.04	215	Unknown		
Maternal smoking in pregnancy, n (%)	87 (40.5%)	211	Unknown		
Preeclampsia-toxemia	53 (24.7%)	214	Unknown		
Treatment with antenatal steroids	124 (57.7%)	215	Unknown		
Breast feeding duration, wks	4.15 ± 0.03	215	Unknown		
*At 28 years*					
Age at Screening, years	28.35 ± 0.07	208	28.18 ± 0.10	94	0.169
Current Smoking, n (%)	65 (30%)	214	20 (21%)	96	0.053
Ever smoked, n (%)	94 (44%)	214	32 (33%)	96	0.051
Diabetes Type 1, n (%)	0 (0%)	215	2 (2.1%)	96	0.095
Diabetes Type 2, n (%)	1 (0.5%)	215	1 (1.0%)	96	0.523
BMI, kg/m^2^	26.95 ± 0.43	205	28.23 ± 0.65	94	0.101
SBP, mmHg	121.85 ± 0.97	207	117.23 ± 1.39	94	**0.008**
DBP, mmHg	75.94 ± 0.72	207	74.02 ± 1.02	94	0.133
MAP, mmHg	87.36 ± 0.67	202	84.76 ± 0.90	94	**0.026**
LVMI, g/m^2^	89.68 ± 1.3	213	95.00 ± 2.31	96	**0.036**
LVEDV, mL/m^2^	58.09 ± 0.74	214	62.40 ± 1.27	96	**0.002**
LVESV, mL/m^2^	20.72 ± 0.33	214	22.60 ± 0.60	96	**0.003**
RV basal dia, cm	3.06 ± 0.03	203	3.23 ± 0.05	96	**0.003**
RAVI, cm^3^/m^2^	25.22 ± 0.46	212	28.36 ± 0.80	96	** < 0.001**
Endo-GLS%	23.66 ± 0.26	108	24.95 ± 0.43	56	**0.008**
CO, L/min	4.92 ± 0.09	209	5.34 ± 0.12	94	**0.009**
SVI, mL/m^2^	37.38 ± 0.48	214	39.81 ± 0.82	96	**0.008**
LnRHI	0.61 ± 0.02	188	0.68 ± 0.03	93	0.062
LV Elastance, mmHg/mL	3.38 ± 0.06	208	2.84 ± 0.08	94	** < 0.001**
Arterial Elastance, mmHg/mL	1.85 ± 0.03	208	1.59 ± 0.04	94	** < 0.001**
FEV1 z-score	− 0.66 ± 0.08	210	− 0.15 ± 0.12	96	** < 0.001**
FEV1 z-score by FVC z-score	− 1.23 ± 0.08	210	− 0.62 ± 0.10	96	** < 0.001**
FEF_25–75_ z-score	− 1.30 ± 0.09	211	− 0.51 ± 0.12	96	** < 0.001**
RV z-score	− 0.81 ± 0.07	212	− 1.22 ± 0.09	96	** < 0.001**
RV by TLCz-score	− 1.06 ± 0.07	212	− 1.49 ± 0.09	96	** < 0.001**
DLCO z-score	− 0.69 ± 0.07	210	− 0.12 ± 0.08	93	** < 0.001**
KCO z-score	− 0.60 ± 0.07	210	− 0.02 ± 0.10	93	** < 0.001**
VO_2_ Max, kg	30.68 ± 0.58	191	31.23 ± 0.83	89	0.590
WBC, 10^9^/L	6.45 ± 0.12	208	6.06 ± 0.17	94	0.066
Platelets 10^9^/L	244.7 ± 4.06	208	252.6 ± 5.90	94	0.278
Neutrophils, 10^9^/L	3.67 ± 0.09	208	3.32 ± 0.13	94	**0.032**
Lymphocytes, 10^9^/L	2.02 ± 0.04	208	2.01 ± 0.05	94	0.893
Monocytes, 10^9^/L	0.52 ± 0.01	208	0.49 ± 0.02	94	0.099
Eosinophils, 10^9^/L	0.22 ± 0.01	205	0.23 ± 0.02	94	0.819
Basophils, 10^9^/L	0.06 ± 0.002	88	0.06 ± 0.002	41	0.299

Data are expressed as mean ± SEM or *n* (%). *p* values <0.005 indicated by bold font

BMI, body mass index; BP, blood pressure; SBP, systolic BP; DBP, diastolic BP; MAP, mean arterial pressure; BSA, body surface area; LVMI, left ventricular mass index; LVEDV, LV end diastolic volume indexed to BSA, LVESV, LV end systolic volume indexed to BSA; RV basal dia, right ventricular basal diameter; RAVI volume, right ventricular volume indexed to BSA; Endo-GLS, Global endocardial longitudinal strain; CO, cardiac output; SVI, Stroke Volume indexed to BSA; LnRHI, Ln Reactive Hyperemia Index; LV Elastance, left ventricular elastance; Z-scores for the following indices: FEV1, forced expiratory volume in the first second; FEF_25–7 5_, forced expiratory flow at 25 and 75% of the pulmonary volume; FEV1 by FVC, ratio of FEV1 to forced vital capacity; RV, residual volume; RV by TLC, ratio of RV to total lung capacity; DLCO, diffusing capacity of the lungs for carbon monoxide; KCO, carbon monoxide transfer coefficient; WBC, white blood cell count

*Controls were considered born at term (37 to 40 weeks) and mean gestation was set at 38.5 weeks

Archived neonatal heel pricks for DNA methylation profiling were only available for a subset of the cohort (109 VLBW, 51 controls), and the characteristics of this subgroup (Additional file 3: Table S1) were similar to the overall cohort, except that differences between VLBW cases and controls in CO, RV basal dia and neutrophils did not reach statistical significance.

For DNA extracted from neonatal blood spots, 93% of samples gave over 80% call rates, with eleven samples having call rates < 80%, (indicating methylation data were obtained from < 80% of the 822,741 valid CpGs on the EPIC arrays for that sample). A total of 12 neonate samples were excluded due to a combination of low call rates and poor red or green channel fluorescence. The remaining neonate samples included in the EWAS had an average call rate of 98% (average 806,286 probes called). For DNA collected from adult peripheral blood samples, Illumina GenomeStudio software indicated all samples had > 99% call rates and high fluorescence intensity on both red and green channels, and hence no samples were excluded due to low quality (822,741 CpG probes included in the EWAS).

### Methylation differences between VLBW cases and controls in neonate samples

**Fig. 1:**
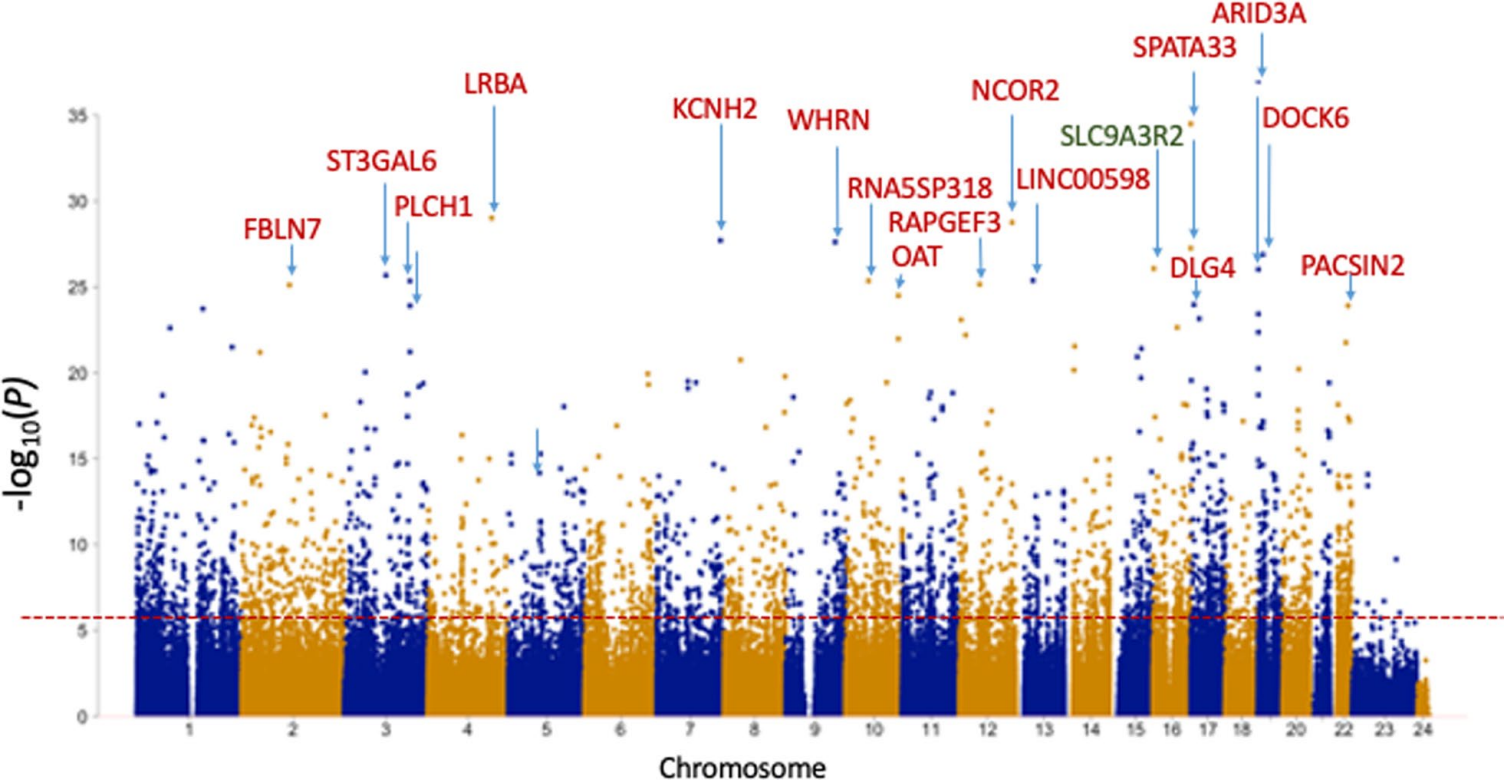
Manhattan Plot of differentially methylated CpGs between VLBW cases and normal birthweight controls in neonatal blood-spot samples (red is hyper-, green is hypomethylated in VLBW cases). Red dotted line indicates FDR-adjusted genome-wide significance *p* < 0.05. Blue arrows indicate the 20 most significant CpGs, with nearest gene name

Analysis of DNA extracted from neonatal blood spots identified over 16,400 CpGs with methylation levels that differed significantly between VLBW and controls (FDR-adjusted *p* < 0.05). The methylation profiles in neonatal samples are illustrated in the Manhattan Plot in Fig. 1, labeled with the names of the nearest gene for the top twenty loci (the full list of CpGs is given in Additional file 4: Table S2 and an interactive Manhattan plot is available at: https://my.locuszoom.org/gwas/506658/?token=2815c5e9b01d4f04a376efb17f9c2f27). The majority of these loci were hypermethylated (Additional file 5: Fig. S1), with a smaller cluster of CpGs being hypomethylated in VLBW cases versus controls.

Gene network analysis performed on the 16,400 CpGs that reached FDR-adjusted significance indicated the canonical pathway most highly enriched with these CpGs was Cardiac Hypertrophy Signaling (*p* = 3.44E^−11^, Additional file 6: Fig. S2), with predicted upregulation of this pathway in VLBW neonates. Physiological functional systems most enriched included Organismal Development and Function (*p* range 2.59E^−07^–3.87E^−27^), Embryonic Development (*p* range 2.04E^−07^–1.24E^−19^) and Cardiovascular System Development and Function (*p* range 1.31E^−07^–1.03E^−18^).

Relationships between neonatal methylation, birthweight and gestation are shown in Table 2 for the twenty CpGs most differentially methylated in VLBW cases versus controls. Nineteen of these CpGs were hypermethylated and one was CpG hypomethylated (cg09476997 in *SLC9A3R2*). The top twenty CpGs included several gene clusters: two CpGs in *ARID3A* (cg02001279 being the most significant CpG overall)*,* two in *SPATA33*, (also known as *C16orf55*) and two in *PLCH1*. Additional CpGs within these same genes also appeared multiple times in the long list of FDR-significant CpGs (Additional file 4: Table S2). Methylation at the majority, but not all, of these CpGs demonstrated significant associations with birthweight and gestational age at birth. The DMR analysis (Additional file 7: Table S3) confirmed that *ARID3A*, *SPATA33*, and *PLCH1*, along with DMRs spanning *MCF2L*, *TRBJ2*-1 and *SRC,* topped the list of 15,000 DMRs reaching FDR-adj significance.

**Table 2:** Top 20 CpGs differentially methylated between VLBW versus controls and associations with birthweight or gestation, adjusting for study group, sex and ethnicity (full list of 16,408 CpGs FDR-adj *p* < 0.05 in Additional file 11: Table S2)

CpG ID and nearest gene	Chromosome: location	*p* value	FDR-adj *p *value*	Fold change	R statistic	Direction in VLBW	Birthweight *p* value and direction (full Cohort)		Gestation *p* value and direction (VLBW cases)	
cg02001279 ***ARID3A***	19:940,967	1.01E^−37^	8.28E^−32^	1.153	0.862	Hypermethylated	0.003	+	< 0.001	+
cg07835443 ***SPATA33/C16orf55***	16:89,734,986	3.17E^−35^	1.30E^−29^	1.217	0.847	Hypermethylated	< 0.001	+	< 0.001	+
cg10461390 ***LRBA***	4:151,324,223	9.63E^−30^	2.64E^−24^	1.154	0.808	Hypermethylated	< 0.001	+	< 0.001	+
cg04347477 ***NCOR2***	12:125,002,007	1.76E^−29^	3.62E^−24^	1.177	0.805	Hypermethylated	0.008	+	< 0.001	+
cg25975961 ***KCNH2***	7:150,600,818	1.97E^−28^	3.24E^−23^	1.144	0.797	Hypermethylated	ns		ns	
cg17307655 ***WHRN/DFNB31***	9:117,214,672	2.42E^−28^	3.31E^−23^	1.143	0.796	Hypermethylated	< 0.001	+	< 0.001	+
cg16725984 ***SPATA33/C16orf55***	16:89,735,184	5.52E^−28^	6.49E^−23^	1.127	0.793	Hypermethylated	0.015	+	< 0.001	+
cg06870470 ***DOCK6***	19:11,315,767	1.31E^−27^	1.35E^−22^	1.167	0.789	Hypermethylated	0.004	+	< 0.001	+
cg09476997 ***SLC9A3R2***	16:2,087,932	8.45E^−27^	7.72E^−22^	0.815	− 0.782	Hypomethylated	0.007	–	0.002	–
cg12713583 ***ARID3A***	19:940,724	9.54E^−27^	7.85E^−22^	1.134	0.781	Hypermethylated	0.012	+	< 0.001	+
cg19274030 ***ST3GAL6***	3:98,489,745	2.12E^−26^	1.58E^−21^	1.116	0.778	Hypermethylated	0.002	+	< 0.001	+
cg00406098 ***LINC00598***	13:40,716,180	4.19E^−26^	2.65E^−21^	1.123	0.775	Hypermethylated	0.001	+	0.006	+
cg06063190 ***RNA5SP318***	10:55,163,525	4.41E^−26^	2.65E^−21^	1.119	0.775	Hypermethylated	0.025	+	0.007	+
cg11932158 ***PLCH1***	3:155,422,129	4.50E^−26^	2.65E^−21^	1.099	0.775	Hypermethylated	ns		0.033	
cg19333758 ***RAPGEF3***	12: 48,135,549	7.05E^−26^	3.87E^−21^	1.166	0.773	Hypermethylated	0.003	+	< 0.001	+
cg19744173 ***FBLN7***	2:112,913,178	7.73E^−26^	3.98E^−21^	1.102	0.773	Hypermethylated	< 0.001	+	< 0.001	+
cg25715278 ***OAT***	10:126,040,305	3.21E^−25^	1.55E^−20^	1.076	0.767	Hypermethylated	0.013	+	< 0.001	+
cg26708048 ***DLG4***	17: 7,087,655	1.08E^−24^	4.94E^−20^	1.121	0.761	Hypermethylated	0.042	+	0.002	+
cg18623216 ***PLCH1***	3:155,421,970	1.24E^−24^	5.12E^−20^	1.102	0.761	Hypermethylated	ns		ns	
cg12405088 ***PACSIN2***	22:43,257,120	1.24E^−24^	5.12E^−20^	1.101	0.761	Hypermethylated	0.049	+	< 0.001	+

^*^ Benjamini–Hochberg FDR *p* value is corrected for 822,741 CpG sites. R statistic is correlation coefficient for continuous variables

We also investigated relationships between methylation at birth and later health outcomes for the twenty most significant CpGs in neonates (using GLMs adjusted for case–control status, sex and ethnicity), with fourteen demonstrating associations with cardiovascular outcomes in young adults aged 28 years (Table 3). Remarkably, twelve of the top CpGs that were different between VLBW cases and controls at birth showed significant associations with adult LnRHI (noting that no associations were observed between methylation in adult samples and LnRHI at any CpG), and the association was especially strong for cg04347477 (*NCOR2)*, cg17307655 (*WHRN)*, cg19333758 (*RAPGEF3)*, cg25715278 (*OAT)* and cg26708048 (*DLG4*). Several CpGs also showed associations between neonatal methylation and systolic BP or indices of cardiac size at 28 years, while methylation of two CpGs, cg25975961 (*KCNH2*) and cg26708048 (*DLG4*), showed associations with adult BMI. Fewer associations between neonatal methylation and adult respiratory traits were observed; the hypomethylated cg09476997 (*SLC9A3R2)* was associated positively with FEV1z by FVCz-score (*p* = 0.037) and negatively with RVz (*p* = 0.012), while the hypermethylated cg11932158 (*PLCH1)* was negatively associated with FEF_25–75_ z-score (*p* = 0.021), FEV1z-score (*p* = 0.024). Other traits not included in Table 3, including a history of smoking, arterial elastance and VO_2_ max, showed no associations between neonatal methylation and adult outcomes.

**Table 3:** CpGs differentially methylated at birth and associations with adult cardiovascular traits at 28 years, adjusting for study group, sex and ethnicity

CpG	Nearest Gene	BMI	Systolic BP	LVMI	LVESV	RV basal dia	RAVI	CO	Endo-GLS	LnRHI
cg02001279	***ARID3A***									0.023
cg07835443	***SPATA33***		0.034							
cg04347477	***NCOR2***								0.016	0.007
cg25975961	***KCNH2***	0.018		^††^ 0.044*				0.041		0.042
cg17307655	***WHRN***					0.048				0.004
cg06870470	***DOCK6***		0.024							0.027
cg09476997	***SLC9A3R2***						0.027^††^	0.009	^††^0.040	
cg12713583	***ARID3A***					0.028				0.033
cg00406098	***LINC00598***			0.046	0.032					0.032
cg06063190	***RNA5SP318***								^††^ 0.035*	0.038
cg19333758	***RAPGEF3***									0.005
cg19744173	***FBLN7***									0.019
cg25715278	***OAT***									0.001
cg26708048	***DLG4***	0.025	0.017							< 0.001

^††^ Preceding the *p* value indicates the interaction term of CpG x Study Group is significant *p* < 0.05; * CpG is significant in VLBW cases when interaction term is also significant in the model

We tested whether potential confounders, including antenatal steroid treatment (ANS), maternal smoking in pregnancy, preeclampsia-toxemia or duration of breastfeeding were accounting for any of these associations with the top 20 CpGs. These variables were tested in neonate cases only (as these data were not available for controls) and weak correlations were identified between maternal smoking and methylation at a single CpG, with preeclampsia-toxemia at four CpGs, with treatment with ANS at three CpGs, and with breastfeeding duration at three CpGs. Adjusting GLM models for the respective potential confounders (in cases only due to lack of data in controls) replicated the previous pattern of associations, albeit with weaker significance due to having only VLBW cases in the model (data not shown). We concluded the associations between top CpGs and adult traits were largely independent of these factors.

### Methylation differences between VLBW cases and controls in adult samples

**Fig. 2:**
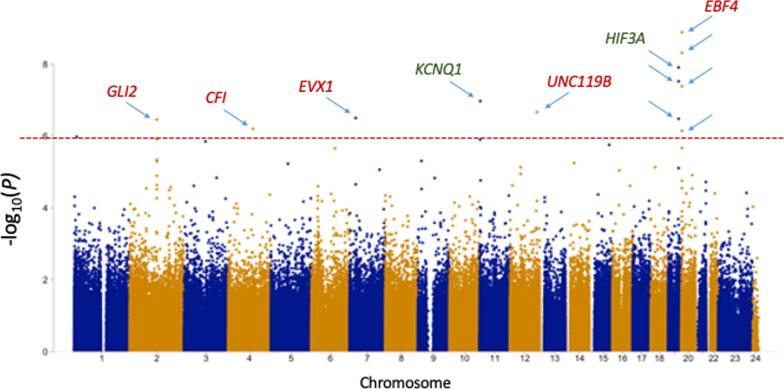
Manhattan plot of CpGs differentially methylated between VLBW cases and normal birthweight controls in samples collected at 28 years of age. The red dotted line indicates the threshold for significance (FDR-adjusted *p* < 0.05), with blue arrows and gene names indicating the 12 CpGs that reached significance (red is hyper-, green is hypomethylated in VLBW cases)

At 28 years of age, twelve CpGs demonstrated altered methylation between VLBW and controls at genome-wide significance (FDR-adj *p* < 0.05), as presented in the Manhattan Plot in Fig. 2 and in Table 4 (an interactive Manhattan Plot is also available at https://my.locuszoom.org/gwas/120785/?token=be8874a3d3374ffba76e7b99fddc2a26). Differentially methylated CpGs included clusters within two genes: four CpGs hypermethylated in *EBF4* and three CpGs hypomethylated in *HIF3A* in VLBW relative to controls, with multiple other CpGs in these two regions also differentially methylated but below our threshold for genome-wide significance. The DMR analysis (Additional file 7: Table S3) confirmed that the multiple CpGs in *HIF3A, EBF4* and *GLI2* were classified as DMRs. Additional CpGs that reached the significance threshold were located in *KCNQ1* (hypomethylated) and *UNC119B, EVX1, GLI2* and *CFI* (all hypermethylated), although cg14486095 in *UNC119B* was determined to be polymorphic and was excluded from further analyses. Methylation levels at the majority of these sites were significantly associated with birthweight, especially CpGs within *EBF4*. Methylation at seven of the lead sites in adults were also weakly associated with gestational age at birth (Table 4, available for VLBW cases only). The DNAm GrimAge scores calculated from the 28-year samples were higher in VLBW cases versus controls (38.12 ± 4.83 versus 36.38 ± 4.87, respectively; *p* = 0.003) as were DNAm GrimAge Adjusted Age scores (0.47 ± 4.77 versus −1.09 ± 4.61, respectively; *p* = 0.007).

**Table 4:** CpGs differentially methylated in adult VLBW versus controls and associations with birthweight or gestation adjusting for study group, sex and ethnicity

CpG ID-nearest gene	Chromosome: location	*p *value	FDR-adj *p* value*	Fold change	*R* statistic	Direction in VLBW	Birthweight *p* value and direction (Full Cohort)		Gestation *p* value and direction (VLBW cases)	
cg13518079 ***EBF4***	20: 2,675,072	1.31E^−09^	0.001	1.067	0.343	Hypermethylated	< 0.001	–	0.015	–
cg05857996 ***EBF4***	20: 2,675,418	4.90E^−09^	0.002	1.065	0.332	Hypermethylated	0.002	–	0.009	–
cg22891070 ***HIF3A***	19: 46,801,642	1.25E^−08^	0.003	0.942	− 0.323	Hypomethylated	0.013	+	0.012	–
cg16672562 ***HIF3A***	19: 46,801,672	3.05E^−08^	0.006	0.944	− 0.315	Hypomethylated	0.015	+	0.013	–
cg24263062 ***EBF4***	20: 2,730,191	4.20E^−08^	0.007	1.047	0.312	Hypermethylated	< 0.001^††^	–	0.011	–
cg26344859 ***KCNQ1***	11: 2,584,629	1.08E^−07^	0.015	0.992	− 0.303	Hypomethylated	ns		ns	
cg14486095 UNC119B**	12: 121,147,222	2.19E^−07^	0.026	1.044	0.296	Hypermethylated	0.028^††^	+	ns	
cg04099095 ***EVX1***	7: 27,278,570	3.19E^−07^	0.029	1.013	0.292	Hypermethylated	0.049	–	ns	
cg27146050 ***HIF3A***	19: 46,801,557	3.35E^−07^	0.029	0.978	− 0.291	Hypomethylated	0.005	+	ns	
cg20219891 ***GLI2***	2: 121,496,875	3.52E^−07^	0.029	1.053	0.291	Hypermethylated	0.027	–	ns	
cg05149986 ***CFI***	4: 110,683,920	6.41E^−07^	0.048	1.015	0.285	Hypermethylated	ns		0.007	–
cg05825244 ***EBF4***	20: 2,730,488	7.23E^−07^	0.049	1.068	0.283	Hypermethylated	< 0.001^††^	–	0.005	–

*Benjamini–Hochberg FDR *p* value is corrected for 822,741 CpG sites. R statistic is correlation coefficient for continuous variables

**cg14486095 in *UNC119B* was found to be polymorphic and was excluded from further analyses

^††^Following the *p* value indicates both the interaction term CpG x Study Group and CpG are significant *p* < 0.05

When pathway analysis was performed on the 1000 most differentially methylated sites in adults (including those below the FDR threshold), the top canonical pathway was Pigment Epithelium-derived Growth Factor (PEDF) signaling (*p* = 1.47E^−04^), but this had only a 9.5% overlap with our list. Gene networks enriched with the adult CpG list included a network featuring upregulation of the gene *HIF3A* and downregulated *Akt,* which was enriched in genes associated with Hereditary Disorders, Neurological Diseases and Organismal Injuries and Abnormalities (Additional file 8: Fig. S3A). A further key network featured downregulation of *EBF4,* predicted inhibition of *Erk1/2*, and was associated with Cellular Development, Embryonic Development, Nervous System Development and Function (Additional file 9: Fig. S4A). The most enriched Physiological Functional System for the adult CpG list was Cardiovascular System Development and Function (*p* range 1.21E^−03^–6.58E^−06^) followed by Organ Morphology (*p* range 1.40E^−02^–8.78E^−06^).

Association between the leading 12 CpGs in adult samples plus Grim Age Adjusted Age score and their concurrent cardiovascular traits (Additional file 10: Table S4A) and respiratory measures (Additional file 10: Table S4B), adjusting for VLBW versus control status, sex and ethnicity in the GLM. Higher methylation levels at *EBF4* CpGs were associated with higher arterial elastance, lower stroke volume (cg05857996 only), RV basal dia or RAVI. Other hypermethylated CpGs also showed associations with smaller indices of cardiac size. The only CpG demonstrating an association with systolic BP was cg05149986 in *CFI*, which was also associated with higher LV elastance and arterial elastance, and with smaller LVMI measurements. The hypomethylated CpGs in *HIF3A* and *KCNQ1* showed associations in the opposite directions to other CpGs, being negatively associated with LV elastance and arterial elastance, and positively associated with (lower) CO and LVMI. Lower methylation at the *KCNQ1* site cg26344859 was also positively associated with lower SVI and smaller LVEDV. Hypomethylation at *HIF3A* sites showed associations with a smaller BMI. DNAm GrimAge Adjusted Age was weakly associated with several cardiovascular measures (Additional file 10: Table S4A). As above, we looked for potential confounders in the 28-year-olds, finding neither treatment with ANS nor beast-feeding duration were correlated with methylation at any CpG, while maternal smoking was correlated with methylation at two CpGs and preeclampsia-toxemia correlated methylation at two CpGs. Adjusting the GLM with these measures in VLBW cases only (due to the lack of data in controls) indicated the associations described above were independent of these confounders.

Additional file 10: Table S4B displays the ten top CpGs that showed any associations with respiratory traits at 28 years. Hypermethylation at *EBF4* was weakly associated with lower DLCOz-scores or lower RVz and RVz by TLC (cg05825244 only). The strongest associations with respiratory traits were between higher methylation at *CFI* and higher RVz by TLC, but lower DLCOz, KCOz and VO_2_ max. Hypomethylation at *HIF3A* generally showed associations with lower RVz and RVz by TLC and higher DLCOz and KCOz. Other CpGs showed associations with other scattered respiratory traits. Across the overall cohort, a history of having ever smoked was associated with methylation at only two CpGs (*HIF3A*, cg22891070 and cg16672562, data not shown, *p* = 0.042 and *p* = 0.023, respectively), both being hypomethylated in smokers. DNAm GrimAge Adjusted Age was strongly associated with several respiratory measures (Additional file 10: Table S4B), and these associations remained even when smoking history was included in the model (data not shown).

### Overlap of CpGs associated with VLBW in both neonate and adult samples

To identify if any CpGs were differentially methylated both at birth and at 28 years of age, regardless of direction, we compared extended lists of CpGs differentially methylated between VLBW and controls (dipping below genome-wide significance in adults). Four CpGs common to both neonate and adult sample lists were identified (Additional file 11: Table S5), including two CpGs in *EBF4* and one in *SNAl1* that were hypomethylated in neonates but hypermethylated in adults, while a CpG in *LOC101928911* was hypermethylated at both ages. At both timepoints, robust associations were observed between these CpGs and several cardiovascular or respiratory variables in adults (see Additional file 11: Table S5), although associations with cardiac dimensions tended to be more marked in controls.

**Fig. 3:**
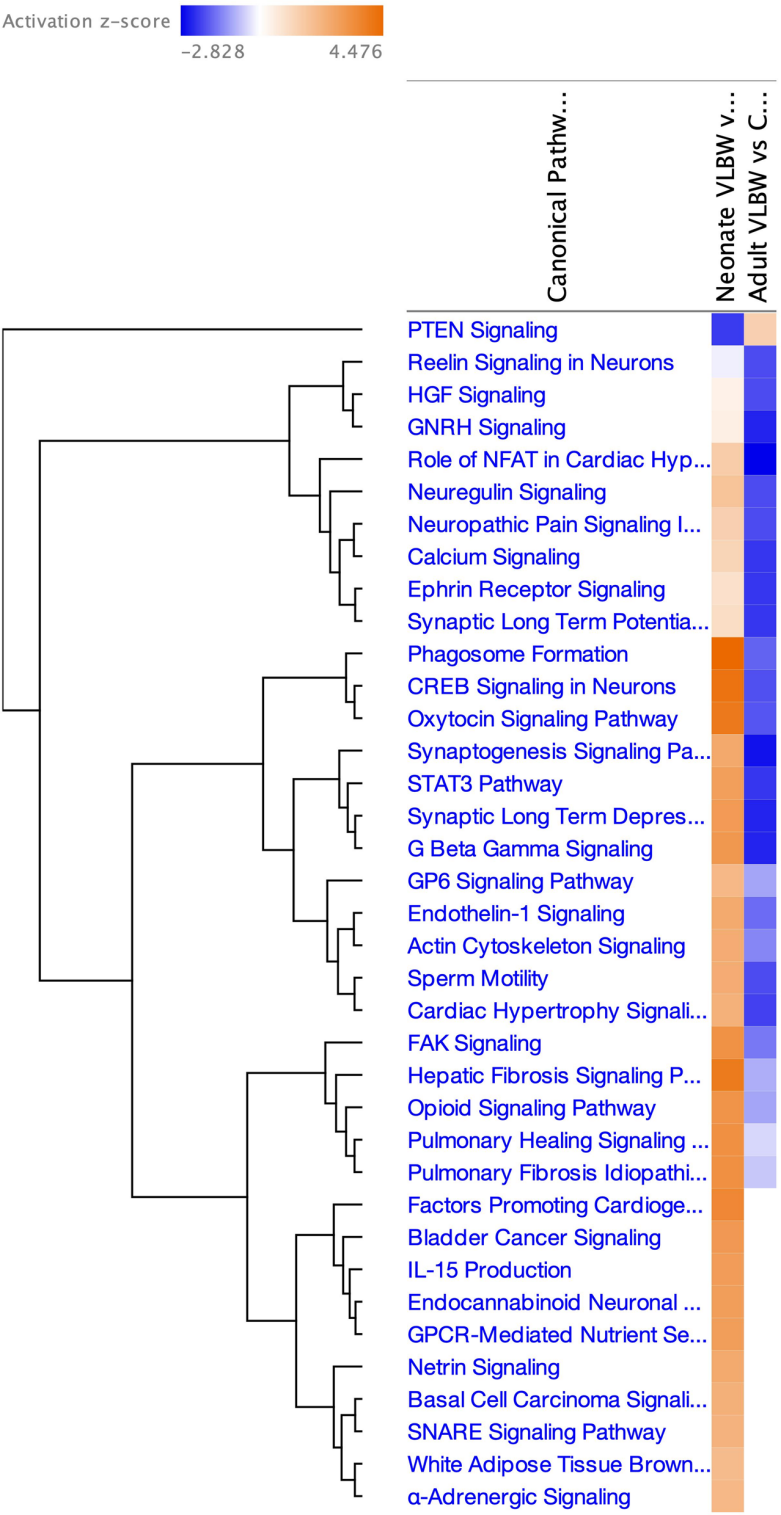
Comparison of Canonical Pathways enriched with CpGs differentially methylated between VLBW cases and controls either in neonates or adults, arranged by hierarchical clustering

To visualize how age-related changes in methylation influenced the activity of gene pathways, we compared canonical pathways enriched for differentially methylated CpGs at each age using Ingenuity Pathway Analysis (IPA, Fig. 3), identifying 27 pathways regulated in opposite directions between birth and adulthood.

We also used IPA to graphically display gene networks enriched for differentially methylated CpGs at each age and to overlay the neonatal methylation data onto the two gene networks identified in the adult samples. The top network from adult data (Additional file 12: Fig. S3B) featured upregulation of *HIF3A* and downregulation of *Akt*, and in neonates a similar pattern of activation and inhibition was demonstrated. In contrast, the second adult gene network featuring *EBF4* (Additional file 13: Fig. S4B) switched direction between neonates and adults: the *EBF4* gene expression changed from strongly upregulated in neonates to downregulated with age, and activation of *Erk1/2* at the network center shifted to inhibition in adults, with associated de-activation of several growth hormone and IGF-binding protein signaling molecules in the network.

We then examined the full methylation dataset in both neonates and adults to identify CpGs showing VLBW-related methylation differences at both ages, irrespective of direction, and this highlighted total of 18 CpGs in *EBF4* that reached genome-wide significance in neonates, with methylation reversing direction in adults although below the genome-wide significance threshold. Principal Component Analysis (PCA) was used to make aggregate methylation scores from these 18 CpGs, performed in both neonate and adult samples separately (Additional file 14: Fig. S5). Associations were observed between adult cardiovascular or respiratory traits and the *EBF4* first and second principal components (PC1 and PC2) at each age (Additional file 15: Table S6). However, associations were generally stronger in controls than in VLBW cases and hence this approach did not add value in predicting outcomes in VLBW babies beyond the top individual CpGs.

### Localization relative to CpG islands and transcription binding sites

Many of our top CpGs were not represented in the DMNIV database, especially those identified in neonate samples, with none of those identified as overlapping enhancer sites. In adult samples the *GLI2* CpG cg20219891 was reported to overlap enhancers, including six enhancers for the genes *GLT2*, *RALB*, *TMEM185B*. Of the top CpGs in neonates, six were located within a CpGs island, four in OpenSea, and the remainder spread across N_Shore, S_Shore or N_Shelf relative to CpG islands. Of the CpGs most significant in adults, 50% of those were shown as being located on a S_Shore relative to a CpG island (including cg16426670 in *EBF4* that was altered in opposite directions in adult and neonate samples), three were within a CpG island, and the remainder spread across N_Shore, S_Shelf or OpenSea.

## Discussion

Individuals in the NZ VLBW cohort are now in their fourth decade and at increased risk of cardiovascular disease and other health sequelae arising from their difficult start in life. Within NZ there is no systematic monitoring of the ongoing health and wellbeing of this population group, nor any mechanism to trigger interventions to prevent the onset of adverse health outcomes. The goal of the current study was to identify methylation markers in neonatal DNA that may be predictive of health outcomes in VLBW adults. This is, to our knowledge, the first report where altered methylation at birth in VLBW infants has been associated with health outcomes as adults, particularly their cardiovascular health. This was consistent with finding that the Cardiac Hypertrophy Signaling pathway was the canonical pathway most highly enriched for CpGs with altered methylation in VLBW neonates. This study is also the first genome-wide screen of DNA methylation sites in adulthood with sufficient statistical power to detect differences between VLBW individuals and controls, highlighting differentially methylated clusters within the *EBF4* and *HIF3A* genes. Moreover, the observation that methylation at *EBF4* CpGs reversed direction between birth and 28 years and was associated with adult outcomes leads us to propose that dynamic methylation of *EBF4* may be associated with the trajectory of compensatory growth and development from birth to adulthood.

We noted many commonalities between our data and that of previous studies of birthweight and methylation, despite differing study designs, such as comparing preterm versus full-term birth rather than birthweight per se, sampling adipose tissue, saliva or cord blood versus peripheral blood, assaying methylation at various time points between birth and middle age, or using differing methylation assay platforms. The finding of analogous methylation sites to our study was particularly striking in a report from Gillberg et al. [60], who examined adult adipose tissue from low- versus normal-birthweight men after 5 days of high-fat diet versus a control diet. They observed 53 CpGs located on 40 genes where methylation differed between low-birthweight men and controls, and of these 40 genes, 21 also featured within our long list of differentially methylated CpGs in neonates (*C17orf97*, *KAZALD1*, *SORBS2*, *DPP10*, *CPLX1, CACNA2D2, FADS2, LOC100271832, CUGBP2, TTYH3, HCCA2, PTPRN2, ACAT1, C7orf50, CASZ1, IGF2R, PDLIM4, CREB3L2, ARHGAP23, ARID1B,* and *UPK3A*).

Findings convergent with the current study can also be seen in the report from Simpkin et al.[52], who performed a longitudinal study of DNA methylation profiles from cord blood and peripheral blood at birth and at ages 7 and 17 years in over 900 children from the Avon Longitudinal Study of Parents and Children (ARIES). They identified 224 CpG sites associated with gestational age (predominantly a negative association, as we also found), and 23 CpG sites associated with birthweight. Their supplementary list of probes where cord blood methylation was negatively associated with gestational age in ARIES showed a remarkable overlap with our most significant CpGs in VLBW neonates, including cg02001279 in *ARID3A*; cg07835443in *C16orf55 (SPATA33)*; cg11932158 in *PLCH1;* cg04347477 in *NCOR2;* cg18623216 in *PLCH1;* cg12713583 in *ARID3A.* Some of the same CpGs were also identified by Cruikshank and co-workers [50], who compared 12 extremely preterm cases and 12 matched controls with DNA from archived blood spots collected from neonates and the same individuals at age 18 years. Although in 18-year-olds no sites achieved genome-wide significance, there was extensive overlap between those CpGs reported by Cruikshank [50] to be different between preterm and term birth and our top CpGs in neonates, including in *C16Orf55* (*SPATA33*), *ARID3A, DOCK6, NCOR2, SLC9A3R2, PLCH1* and *FBLN7*.

A meta-analysis of epigenome-wide association studies with birthweight by Küpers et al. [37], included 8825 neonates from 24 birth cohorts in the Pregnancy And Childhood Epigenetics Consortium and demonstrated that lower birthweight, even within the normal range, is related to altered DNA methylation at 914 sites. In additional analyses in 7278 participants, < 1.3% of birthweight-associated differential methylation was also observed in childhood and adolescence, and this study could find no associations in adulthood (30–45 years). The top hits for association with birthweight included some minor overlap with gene families we observed associated with VLBW in neonates, such as *ARID5B ARHGAP20*, *ARHGAP29*, and *ARHGAP45*. However, Küpers and colleagues [37] also identified 147 birthweight-related methylation quantitative trait loci (mQTL, where genetic variants are associated with methylation), which included 23 CpGs from our neonate long list, most notably cg06870470 in *DOCK6*, one of our most significant CpGs at birth.

Other prior studies that did not replicate the current findings include a report from Tan et al. [61], who carried out DNA methylation profiling of preterm birth in 144 adult twins with a median age of 33 years (with 26 twin pairs of premature birth). They found three genomic regions associated with preterm birth annotated to the *SDHAP3, TAGLN3 (*both hypomethylated) and *GSTT1* (hypermethylated) genes. While none of these CpGs reached genome-wide significance individually, combining multiple CpGs in each locus led to the clusters attaining significance. These differentially methylated regions replicated in an older, independent set of 175 twin pairs (median age 66 years) with 40 twins classified as preterm birth (at least 3 weeks before term). Finally, Wheater et al*.* [62] investigated the impact of low gestational age at birth on methylation patterns in neonatal saliva samples and also documented associations of methylation with brain white matter structure by diffusion magnetic resonance imaging of these infants*.* The most significant probes associated with gestation in the Wheater study showed no apparent overlap with CpGs identified in neonates in this current study, possibly related to their use of saliva samples versus peripheral blood.

In this current study, we observed age-related changes in methylation at *EBF4*. Between birth and 28 years, the CpG that showed the greatest reversal in direction of altered methylation in VLBW cases compared to controls (*EBF4* cg16426670) was associated with cardiovascular traits in adulthood. The *EBF* gene belongs to a family of transcription factors associated with B-lymphocyte maturation and neuronal development, and also has a role in governing the differentiation of cardio-pharyngeal mesoderm into heart versus pharyngeal muscle fate [63]. Methylation of members of this gene family have been associated with a number of phenotypes, including *EBF4* methylation associated with hematopoiesis and neuronal development in persons with Trisomy 21 [64], and *EBF1* and *EBF3* methylation to neurobehavioral development in very preterm infants [65]. Our observation of dynamic methylation of *EBF4* between birth and adulthood extends the findings of Simpkin and colleagues [52], who reported dynamic *EBF4* methylation in children up to 7 years of age. Their analysis of serial methylation from birth to adolescence suggested that methylation differences did not persist beyond early childhood, with the authors suggesting that methylation levels had largely stabilized by age 7. Notably, however, that study found that among 36 probes with increased methylation per week of gestation, the probe with the maximum increase during childhood was cg16426670 in *EBF4,* showing 6.7% increase per year between birth and 7 years, the same CpG probe that we found to switch from hypomethylation in VLBW neonates to hypermethylation in VLBW adults compared with controls.

A recent study by Long et al*.* [66] investigated associations between methylation at copper-related CpGs and risk of acute coronary syndromes. They found higher methylation at cg05825244 in *EBF4* (among our 12 CpGs differentially methylated in VLBW adults), which was associated with a 23% increased risk of acute coronary syndromes. Further, mass spectrometry of sera from patients about to undergo coronary artery bypass graft (CABG) surgery identified that presence of EBF4 protein was more prevalent and helped distinguish patients with T2DM [67]. Our gene network analysis identified that in VLBW adults *EBF4* is a key gene in a network centering on the serine/threonine protein kinase, *Akt*, which has key roles in many signaling pathways [68]. Together, these findings suggest that the reversal from *EBF4* hypomethylation in VLBW neonates to hypermethylation in adulthood may be involved in the compensatory catch-up of growth and development of the cardiovascular system. It is plausible that overcompensation of the *EBF4* pathway with age may even contribute to adult cardiovascular risk in VLBW cases, with lower *EBF4* methylation levels at birth potentially associated with higher adult systolic blood pressure, smaller cardiac dimensions and lower cardiac output. Future functional studies in animal models may be needed to clarify the relationship between *EBF4* methylation and phenotypic traits of adult VLBW survivors.

Methylation patterns have been used to generate epigenetic age estimates, which have now been established to predict chronic disease burden and time to death [48, 49]. Using the DNAm GrimAge calculator, we confirmed previous reports that VLBW survivors have significantly greater methylation age scores than age-matched controls. However, the GrimAge scores for both cases and controls (38.12 versus 36.38 years, respectively) indicate a greater age than the participants’ chronological age. We suggest the reason may be that these algorithms were derived to predict chronic disease mortality risk in middle age and they may not work as well in younger individuals. However, in this study we compared like-with-like, since the VLBW cases and controls were of equivalent ages at sample collection. We have provided the GrimAge data as a useful data reduction tool, to combine multiple methylation markers previously associated with mortality risk, rather than an accurate predictor of an individual’s biological age.

Van Lieshout et al.[53] compared epigenetic age estimates in adults aged 30 to 35 years born extremely low birthweight (ELBW) (< 1000 g), with a sample of age- and sex-matched controls. This study analyzed DNA from buccal cells and generated a methylation score consisting of 353 CpGs in Horvath’s epigenetic-clock algorithm. They demonstrated that ELBW men had a significantly older epigenetic age (an additional 4.6 years) than normal birthweight men, although women born ELBW were not found to be epigenetically older than their normal birthweight peers. A further study of 143 ELBW infants born 1991–1992 in Victoria, Australia, used DNA extracted from neonatal blood spots collected after birth to generate an algorithm to estimate DNAm-based gestational age [51]. The residual of DNAm gestational age and clinically estimated gestational age (referred to as “gestational age acceleration”) was used to assess developmental maturity. Infants with higher gestational age acceleration were less likely to have received surfactant or postnatal corticosteroids, had fewer days of assisted ventilation, and less frequently had bronchopulmonary dysplasia.

There are several limitations of our study. Firstly, DNA methylation analysis in neonates was performed on dried blood spots that had been archived for 30 years, while in the young adults we were able to extract DNA from recently collected whole blood. The differences in storage time and DNA sample preparation method meant that it would be inappropriate to directly compare raw methylation data files between ages; rather we analyzed the samples separately and compared the resultant lists of candidate CpGs with differing methylation between VLBW cases and controls. Secondly, data relating to pregnancy and the perinatal period was only available for VLBW cases, and not available for controls. Consequently, associations between methylation and potential confounders such as maternal smoking in pregnancy, preeclampsia-toxemia, treatment with antenatal steroids and duration of breast feeding could only be explored in VLBW cases, not analyzed over the entire cohort.

In future work, it would be of interest to relate our findings to current cardiovascular and respiratory health outcomes of the Victoria cohort [50, 51], and to examine the associations with their neonatal DNAm gestational age. Those infants were born 1991–1992 and would now be aged 29–30 years, similar to the age of the NZ VLBW cohort in the present study.

## Conclusions

Levels of DNA methylation in samples collected at birth from VLBW infants have altered methylation that is predicted to lead to perturbations of underlying gene signaling pathways involved in cardiovascular development and hypertrophy. Moreover, the altered methylation profiles at birth show associations with cardiovascular and respiratory health in adulthood, with dynamic methylation in *EBF4* (especially cg16426670) potentially informative for cardiovascular outcomes in later life. Thus, methylation patterns in VLBW neonates and young adults, in combination with clinical data collected at birth beyond birthweight and gestational age, may provide predictive information of those individuals at risk of future adverse health outcomes.

## Supplementary Information

**Additional file 1: Methods 1.** Flow chart of the New Zealand 1986 VLBW Cohort follow-up study recruitment process, with inclusion and exclusion criteria for DNA methylation of samples collected at birth and at 28 years.

**Additional file 2: Methods 2**. The modified protocol used for extraction of DNA from archived neonatal dried blood spots and the method for bisulphite conversion used prior to analysis on Human MethylationEPIC 850K arrays.

**Additional file 3: Table S1**. Characteristics of the subset of VLBW cases and controls with neonatal DNA available.

**Additional file 4: Table S2**. Full list of 16407 CpGs with methylation that differed between VLBW cases and controls after adjusting for false discovery rate (Benjamini-Hochberg FDR adj-*p*<0.05).

**Additional file 5: Figure S1**. Image of the CpGs with significantly different methylation between VLBW cases and controls in neonate samples; hypermethylated clustered on the right and hypomethylated CpGs clustered on the left (also enlarged in the inset, indicating CpGs within *EBF4* that reached FDR-adj significance).

**Additional file 6: Figure S2**. In neonatal DNA the top canonical pathway enriched with the CpGs different between VLBW cases and controls was Cardiac HypertrophySignaling (*p* = 3.44E^−11^).

**Additional file 7: Table S3**. Differentially methylated regions (DMRs) that were significant (FDR adj-*p*<0.05), with DMRs in neonatal samples on the first worksheet and DMRs identified in adult samples on the second worksheet.

**Additional file 8: Figure S3a**. In adult samples, the gene network most enriched for CpGs differentially methylated in VLBW cases versus controls, which predicted upregulation of *HIF3A* gene expression and downregulation of *Akt*, was associated with Hereditary Disorders, Neurological Diseases and Organismal Injuries and Abnormalities.

**Additional file 9: Figure S4a**. In adult samples, a further gene network enriched for CpGs differentially methylated in VLBW cases versus controls, which predicted downregulation of *EBF4* and inhibition of *Erk1/2*, was associated with Cellular Development, Embryonic Development, Nervous System Development and Function.

**Additional file 10: Tables S4a and S4b**. Associations between CpGs with differential DNA methylation in adult samples, DNAm GrimAge and cardiovascular variables (4A) or respiratory traits (4B) at 28 Years.

**Additional file 11: Table S5**. From the lists of CpGs with differential DNA methylation in VLBW cases versus controls, four overlapped in both neonates and adults and showed several associations with adult cardiovascular and respiratory traits.

**Additional file 12: Figure S3b**. Overlay of neonatal methylation data onto the top gene network identified in adult samples, showing a similar pattern of predicted upregulation of *HIF3A* gene expression and downregulated *Akt* in both neonate and adult samples for this gene network.

**Additional file 13: Figure S4b**. Overlay of neonatal methylation data onto the second gene network identified in adult samples, showed this network switched direction with age: *EBF4* gene expression changed from strongly upregulated in neonates to downregulated in adults, activation of *Erk1/2* at the center of the network shifted to inhibition, with associated de-activation of several growth hormone and IGF-binding protein signaling molecules in the network.

**Additional file 14: Table S5**. Principal Component Analysis (PCA) of the 18 CpGs in *EBF4* with differential methylation in both neonate and adult datasets, irrespective of direction.

**Additional file 15: Table  S6**. After PCA was performed on the 18 *EBF4* CpGs identified from both adult and neonate methylation data, associations were observed between the first and second principal components (PC1 and PC2) at each age and adult cardiovascular or respiratory traits.

## Data Availability

The data that support the findings of this study are not openly available due to privacy considerations for this small cohort (all infants born VLBW in NZ in 1986) and the sensitivity of data from Indigenous Peoples, with 30% of the cohort identifying as Māori. However, the DNA methylation summary data are available through the following links. VLBW neonate: https://my.locuszoom.org/gwas/506658/?token=2815c5e9b01d4f04a376efb17f9c2f27. VLBW adult: https://my.locuszoom.org/gwas/120785/?token=be8874a3d3374ffba76e7b99fddc2a26.
