# Supplementary material for: DNA methylation patterns at birth predict health outcomes in young adults born very low birthweight
Source: Clin Epigenetics. 2023 Mar 23;15:47. doi: 10.1186/s13148-023-01463-3 (PMC10035230; doi:10.1186/s13148-023-01463-3)
Supplement: Supplementary file 1 — Additional file 1: Methods 1. Flow chart of the New Zealand 1986 VLBW Cohort follow-up study recruitment process, with inclusion and exclusion criteria for DNA methylation of samples collected at birth and at 28 years. [file 13148_2023_1463_MOESM1_ESM.pdf]

New Zealand 1986 VLBW Adult Follow-up Study: Cohort flow chart

Recorded as live born with birth weight <1500g and admitted to a neonatal unit in New Zealand in 1986
n=413

↓

Included in prospective cohort audit of retinopathy of prematurity
n=413

→ Died before discharge from NICU *n*=75
Died between discharge and 7 years *n*=12

↓

Alive at 7 years and eligible for follow-up *n*=326

ASSESSED at 7-8 years at a home visit[a]	*n*=298	NOT ASSESSED at age 7-8 years	*n*=28
		Overseas/ not approached	*n*=17
		Lost to follow-up	*n*=4
		Declined	*n*=7

→ Died between 8 and 22 years *n*=3

↓

Alive at 22 years and eligible for follow-up *n*=323

ASSESSED at 22-23 years at face-to-face interview[a]	*n*=230	NOT ASSESSED at 22-23 years	*n*=93
		Not traced	*n*=72
		Declined	*n*=21

→ Died between 22 and 26 years *n*=0

↓

Alive at 26 years and eligible for follow-up *n*=323

ASSESSED at 26-30 years	*n*=250	NOT ASSESSED at 26-30 years	*n*=73
Full assessment over 2 days[b]		Not traced	*n=35*
(including 29 living overseas)	*n*=229	Declined	*n=38*
Assessment by questionnaire only[b]	*n*=21		
**DNA available for methylation**	***n=215***		

[a]At 7 to 8 and 22 to 23 years of age, pulmonary function tests were not undertaken. [b]Between February 2013 and November 2016 NICU: Neonatal intensive care unit

[Modified, with permission from Darlow BA, et al. Metabolic syndrome in very low birth weight young adults and controls: the New Zealand 1986 VLBW Study. *J Pediatr* 2019;206:128-33 (133.e1)]
