# Supplementary material for: DNA methylation patterns at birth predict health outcomes in young adults born very low birthweight
Source: Clin Epigenetics. 2023 Mar 23;15:47. doi: 10.1186/s13148-023-01463-3 (PMC10035230; doi:10.1186/s13148-023-01463-3)
Supplement: Supplementary file 2 — Additional file 2: Methods 2. The modified protocol used for extraction of DNA from archived neonatal dried blood spots and the method for bisulphite conversion used prior to analysis on Human MethylationEPIC 850K arrays. [file 13148_2023_1463_MOESM2_ESM.docx]

**VLBW NEONATAL DRIED BLOOD SPOT DNA EXTRACTION PROTOCOL**

**Maximum of 12 Samples per batch**

**Material:**

- QIAamp® 96 DNA Blood Kit (Cat # 51161)
- Buffer ATL (warm in water bath at 50^o^C for 5 min if cloudy)
- Proteinase K
- Ice-cold 100% ethanol (keep in freezer, need 24 ml per batch)
- Ice-cold 80% ethanol (keep in freezer, need 36 ml per batch)

**Preparation:**

For every patient sample set (ie 3 x 3mm DBS), label 4 sets of 2 mL tubes with study ID on tube lid and side: Tube A, B and C with brief label, Tube D with full label.

- Tube A: contains DBS
- Tube B: for pooling supernatant from repeated ATL incubation
- Tube C: parallel alcohol precipitation
- Tube D: parallel alcohol precipitation

**Method:**

- Add 300 µL of Buffer ATL into tube A containing 3 DBS pieces, incubate overnight at 37°C with shaking at 500 rpm (warm Buffer ATL in water bath at 50^o^C for 5 min if cloudy)

WAIT TIME: OVERNIGHT ~19 HRS

1. Day 2 – Set water bath at 85°C
2. Incubate tube A at 85°C for 10 min in water bath
3. Transfer supernatant to tube B (leaving behind DBS in tube A)
4. Add 190 µL of Buffer ATL into tube A, incubate at 85°C for 10 min
5. Transfer supernatant to tube B (leaving behind DBS in tube A)
6. Add 190 µL of Buffer ATL into tube A, incubate at 85°C for 10 min
7. Move whole volume in tube B back to tube A
8. Add 75 µL of Proteinase K, shake the tubes vigorously and incubate on heatblock at 56°C for 1 hours (no shaking)
9. Set water bath temperature to 70°C, open lid to help temperature drop

WAIT TIME: 1 HOUR

1. Add 750 µL of Buffer AL to tube A, invert to mix
2. Incubate tubes in waterbath at 70°C for 10 min
3. While waiting, add 1 mL of ice cold 100% ethanol to tube C and tube D, keep tubes in freezer until ready
4. At the end of Buffer AL incubation, spin the tubes briefly
5. Take tubes C and D out from freezer, add ~755ul tube A to each of tube C and tube D
6. Invert to mix, put into -30°C freezer for 1 hour
7. Set centrifuge to 4°C after putting samples in freezer

WAIT TIME: 1 HOUR

1. Spin the tube at maximum speed at 4°C for 30 min (hinge side out)

WAIT TIME: 0.5 HOUR

1. Carefully tip off ethanol, and wash the pellet with 1.5 mL of ice cold 80% ethanol
2. Invert tubes to mix and spin the tube at maximum speed at 4°C for 30 min (hinge side out)

WAIT TIME: 0.5 HOUR

1. Remove supernatant in tube C & D using pipette, then add 100 ul nuclease-free water to tube C. Dissolve DNA pellet by doing 30 x up & downs (pellet usually invisible but should be at bottom of tube on hinge side)
2. Aspirate 100 ul from tube C, dispense into tube D to dissolve DNA pellet, ~30 up & downs
3. Spin down and dry in vacuum concentrator for 50 min at 30°C

WAIT TIME: ~1 HOUR

1. Dissolve DNA pellet in 25 ul Buffer AE
2. Measure DNA quantity and 260:280 ratio on Nanodrop (ThermoFisher Scientific, Waltham, MA, USA)

**BISULPHITE CONVERSION PROTOCOL***

Using Zymo EZ-96 Methylation Kit (Shallow-Well Format) D5003

* Note: This method differs from manufacturer’s brief protocol, see page 6 of full Zymo manual (D5004).

1. Prepare reagents as per kit protocol
2. Add M-dilution to each DNA sample in the conversion plate and adjust volume to 50 uL with water as per instructions, to make 1000 uL total.
3. Incubate conversion plate at 37 ^o^C for 15 mins in thermal cycler.
4. Add 100 uL prepared CT reagent to each sample and mix by pipetting up and down. Do as quickly as possible to prevent CT reagent from degrading in the light (Do not use foil seals provided as these come apart in thermal cycler).
5. Incubate in thermal cycler (95 ^o^C 30 secs / 50 ^o^C 60 mins) x 16 cycles.* Hold at 4 ^o^C for 10 mins.
6. After incubation spin plate briefly, then add M-binding buffer as per instructions. Pipette samples up and down to mix and transfer to M-binding plate as per instructions.
7. Follow protocol but elute in 25 uL rather than 30 uL.
