# Supplementary material for: DNA methylation patterns at birth predict health outcomes in young adults born very low birthweight
Source: Clin Epigenetics. 2023 Mar 23;15:47. doi: 10.1186/s13148-023-01463-3 (PMC10035230; doi:10.1186/s13148-023-01463-3)
Supplement: Supplementary file 3 — Additional file 3: Table S1. Characteristics of the subset of VLBW cases and controls with neonatal DNA available. [file 13148_2023_1463_MOESM3_ESM.docx]

**Supplementary Table 1: Characteristics of VLBW Cases and Controls with Neonatal DNA Available**

|  | | **VLBW** | **n** | **Controls** | **n** | ***p* value** |
| --- | --- | --- | --- | --- | --- | --- |
| **At Birth** | | | | | | |
|  | Birth Weight, g | 1153 ± 21.55 | 109 | 3530 ± 71.32 | 51 | **<0.001** |
|  | Female n (%) | 66 (61%) | 109 | 33 (65%) |  | 0.373 |
|  | Gestation at birth, weeks | 29.06 ± 0.23 | 109 | 38.50 ± 0 | 51 | **<0.001** |
|  | Ethnicity  Māori  Pacific  European | 30 (27.5%)  2 (1.8%)  77 (70.6%) |  | 6 (11.8%)  4 (7.8%)  41 (80.4%) |  | **0.022** |
|  | Ventilation, days | 9.64 ± 1.33 | 109 | unknown | - |  |
|  | Maternal Smoking in pregnancy, n (%) | 44 (41%) | 107 | unknown | - |  |
|  | Preeclampsia-toxemia | 20 (18.3%) | 109 | unknown | - |  |
|  | Treatment with antenatal steroids | 64 (58.7%) | 109 | unknown | - |  |
|  | Breast feeding duration, wks | 4.37 ± 0.56 | 104 | unknown | - |  |
| **At 28 Days** | | | | | | |
|  | Age at screening, years | 28.40 ± 0.11 | 104 | 28.20 ± 0.14 | 51 | 0.280 |
|  | Current Smoking, n (%)  Ever smoked, n (%) | 29 27%  49 (45%) | 109  109 | 8 (16%)  13 (25%) | 51  51 | 0.090  **0.014** |
|  | Diabetes Type 1, n (%) | 0 (0%) | 109 | 0 (0%) | 51 | - |
|  | Diabetes Type 2, n (%) | 1 (<1%) | 109 | 0 (0%) | 51 | 0.681 |
|  | BMI, kg/m^2^ | 27.45 ± 0.58 | 104 | 27.53 ± 0.78 | 51 | 0.934 |
|  | Systolic BP, mmHg | 121.69 ± 1.37 | 103 | 116.14 ± 1.92 | 51 | **0.020** |
|  | Diastolic BP, mmHg | 75.45 ± 1.00 | 103 | 72.96 ± 1.23 | 51 | 0.130 |
|  | MAP, mmHg | 86.30 ± 0.93 | 101 | 84.75 ± 0.91 | 51 | 0.288 |
|  | LVMI indexed to BSA, g/m^2^ | 88.45 ± 1.84 | 109 | 96.54 ± 25.8 | 51 | **0.028** |
|  | LVEDV indexed to BSA, mL/m^2^ | 58.97 ± 1.07 | 109 | 63.88 ± 1.84 | 51 | **0.016** |
|  | LVESV indexed to BSA, mL/m^2^ | 21.03 ± 0.50 | 109 | 23.13 ± 0.87 | 51 | **0.026** |
|  | RV basal diameter, cm | 3.09 ± 0.04 | 100 | 3.24 ± 0.07 | 50 | 0.060 |
|  | RAVI, cm^3^/m^2^ | 24.54 ± 0.62 | 109 | 29.02 ± 1.17 | 51 | **<0.001** |
|  | Global endocardial longitudinal strain% | 23.22 ± 0.39 | 51 | 24.96 ± 0.63 | 28 | **0.016** |
|  | Cardiac Output, L/min | 5.04 ± 0.13 | 104 | 5.35 ± 0.17 | 51 | 0.165 |
|  | Stroke Volume indexed to BSA, mL/m^2^ | 37.94 ± 0.69 | 109 | 40.74 ± 1.14 |  | **0.029** |
|  | LnRHI | 0.63 ± 0.03 | 95 | 0.67 ± 0.04 | 49 | 0.390 |
|  | LV Elastance, mmHg/mL | 3.28 ± 0.08 | 104 | 2.77 ± 0.10 | 51 | **<0.001** |
|  | Arterial Elastance, mmHg/mL | 1.79 ± 0.04 | 104 | 1.55 ± 0.06 | 51 | **<0.001** |
|  | FEV1 z-score | -0.64 ± 0.12 | 108 | -0.15 ± 0.15 | 51 | **0.017** |
|  | FEV1 z-score by FVC z-score | -1.21 ± 0.11 | 108 | -0.57 ± 0.13 | 51 | **<0.001** |
|  | FEF_25–75_ z-score | -1.31 ± 0.13 | 108 | -0.48 ± 0.16 | 51 | **<0.001** |
|  | RV z-score | -0.82 ± 0.99 | 108 | -1.20 ± 0.12 | 51 | **0.021** |
|  | RV by TLCz-score | -1.09 ± 0.09 | 108 | -1.45 ± 0.10 | 51 | **0.019** |
|  | DLCO z-score | -0.70 ± 0.10 | 107 | -0.16 ± 0.11 | 49 | **0.002** |
|  | KCO z-score | -0.55 ± 0.10 | 107 | 0.01 ± 0.14 | 49 | **0.002** |
|  | VO_2_ Max, kg | 30.10 ± 0.87 | 89 | 32.37 ± 1.14 | 46 | 0.122 |
|  | WBC, 10^9^/L | 6.30 ± 0.15 | 104 | 5.93 ± 0.24 | 51 | 0.178 |
|  | Platelets 10^9^/L | 242.63 ± 5.37 | 104 | 248.31 ± 7.97 | 51 | 0.550 |
|  | Neutrophils, 10^9^/L | 3.49 ± 0.11 | 104 | 3.20 ± 0.19 | 51 | 0.154 |
|  | Lymphocytes, 10^9^/L | 2.05 ± 0.06 | 104 | 2.01 ± 0.07 | 51 | 0.680 |
|  | Monocytes, 10^9^/L | 0.51 ± 0.02 | 104 | 0.47 ± 0.02 | 51 | 0.145 |
|  | Eosinophils, 10^9^/L | 0.22 ± 0.02 | 103 | 0.22 ± 0.02 | 51 | 0.828 |
|  | Basophils, 10^9^/L | 0.06 ± 0.003 | 50 | 0.06 ± 0.004 | 29 | 0.285 |

Data are expressed as mean ± sem or n (%). Abbreviations: BMI, body mass index; BP, blood pressure; MAP, mean arterial pressure; BSA, body surface area; LVMI, left ventricular mass; LVEDV, LV end diastolic volume, LVESV, LV end systolic volume; RV basal diameter, right ventricular basal diameter; RA volume, right ventricular volume; LV Elastance, left ventricular elastance; Z-scores for the following indices: FEV1, forced expiratory volume in the first second; FEF_25–7 5_, forced expiratory flow at 25 and 75% of the pulmonary volume; FEV1 by FVC, ratio of FEV1 to forced vital capacity; RV, residual volume; RV by TLC, ratio of RV to total lung capacity; DLCO, diffusing capacity of the lungs for carbon monoxide; KCO, carbon monoxide transfer coefficient; WBC, white blood cell count.
