# Supplementary figures and images for: DNA methylation patterns at birth predict health outcomes in young adults born very low birthweight
Source: Clin Epigenetics. 2023 Mar 23;15:47. doi: 10.1186/s13148-023-01463-3 (PMC10035230; doi:10.1186/s13148-023-01463-3)

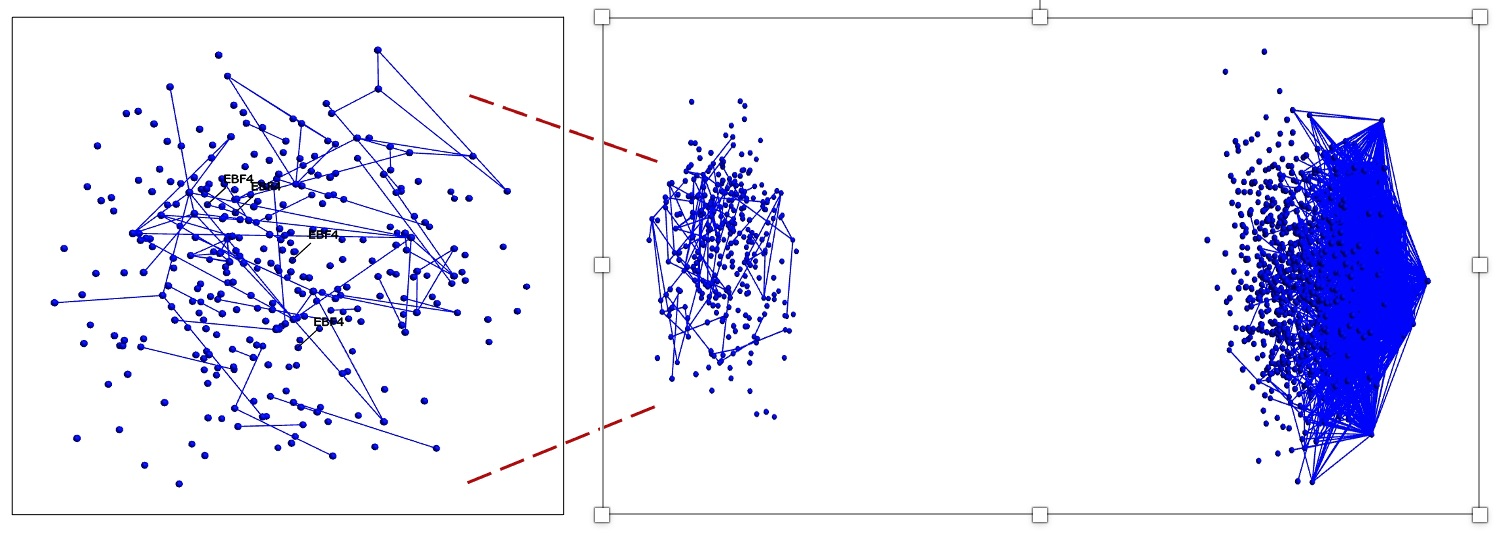

Supplement: Supplementary file 5 — Additional file 5: Figure S1. Image of the CpGs with significantly different methylation between VLBW cases and controls in neonate samples; hypermethylated clustered on the right and hypomethylated CpGs clustered on the left (also enlarged in the inset, indicating CpGs within EBF4 that reached FDR-adj significance). [file 13148_2023_1463_MOESM5_ESM.tiff]

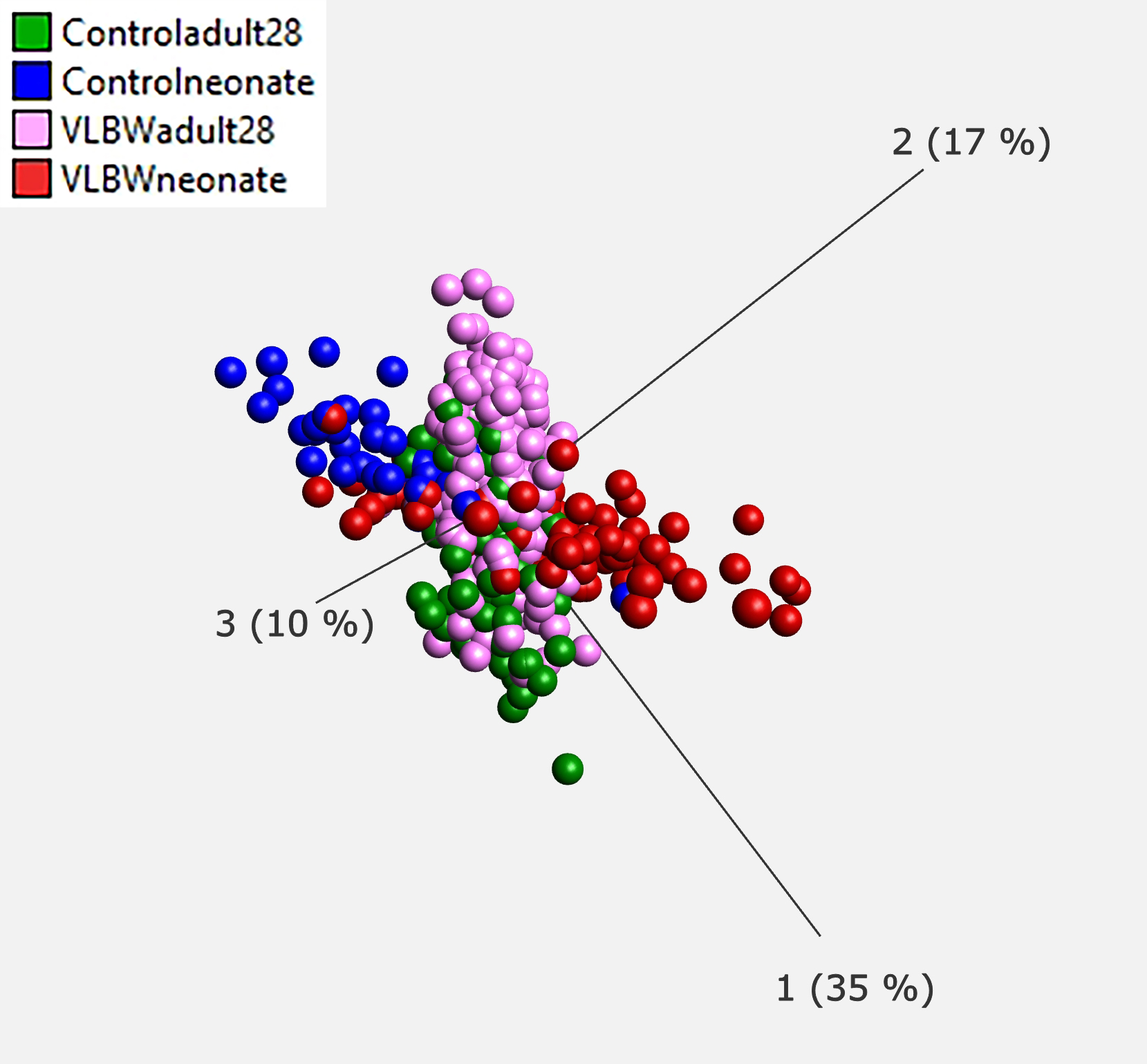

Supplement: Supplementary file 14 — Additional file 14: Table S5. Principal Component Analysis (PCA) of the 18 CpGs in EBF4 with differential methylation in both neonate and adult datasets, irrespective of direction. [file 13148_2023_1463_MOESM14_ESM.tiff]
