# Supplementary material for: DNA methylation patterns at birth predict health outcomes in young adults born very low birthweight
Source: Clin Epigenetics. 2023 Mar 23;15:47. doi: 10.1186/s13148-023-01463-3 (PMC10035230; doi:10.1186/s13148-023-01463-3)
Supplement: Supplementary file 10 — Additional file 10: Tables S4a and S4b. Associations between CpGs with differential DNA methylation in adult samples, DNAm GrimAge and cardiovascular variables (4A) or respiratory traits (4B) at 28 Years. [file 13148_2023_1463_MOESM10_ESM.docx]

**Supplementary Table 4A: CpGs with Differential DNA Methylation at 28 Years and Associations with Adult Cardiovascular Traits, Adjusting for Study Group, Sex and Ethnicity**

| **CpG -**  **Gene** | **BMI** | **Systolic BP** | **LVMI**  **Indexed to BSA** | **LVEDV Indexed to BSA** | **LVESV Indexed to BSA** | **Stroke Vol Indexed to BSA** | **Cardiac Output** | **LV**  **Elastance** | **Arterial Elastance** | **RV Basal Diameter** | **RAVI** |
| --- | --- | --- | --- | --- | --- | --- | --- | --- | --- | --- | --- |
| cg13518079  ***EBF4*** |  |  |  |  |  |  |  |  | 0.015 | 0.016 | 0.018 |
| cg05857996  ***EBF4*** |  |  |  |  |  | 0.029 |  |  | 0.026 | 0.015 | ^††^ 0.040 |
| cg22891070  ***HIF3A*** | 0.004 |  | 0.039 |  |  |  | 0.026 | 0.046 | 0.013 |  |  |
| cg16672562  ***HIF3A*** | 0.018 |  | 0.013 |  |  |  | 0.045 | 0.041 | 0.004 |  |  |
| cg26344859  ***KCNQ1*** |  |  |  | 0.023 |  | 0.020 |  |  |  |  |  |
| cg14486095  ***UNC119B*** |  |  |  |  |  |  |  |  |  | 0.045 |  |
| cg27146050  ***HIF3A*** |  |  | 0.007 |  |  |  |  |  | 0.011 |  |  |
| cg20219891  ***GLI2*** |  |  |  | 0.045 | 0.038 |  |  |  |  |  | 0.018 |
| cg05149986  ***CFI*** |  | 0.039 | 0.008 |  |  |  |  | 0.048 | 0.016 |  |  |
| cg05825244  ***EBF4*** |  |  |  |  |  |  |  |  |  | 0.023 | 0.038 |
| **DNAm GrimAge Adjusted Age** |  |  | 0.013^††^ | 0.032 |  | 0.036 |  |  | 0.041 |  |  |

*p* values for positive associations are in red and negative associations in blue. CpGs not included in the Table were not significantly associated with any trait. ^††^ Preceding the p-value indicates there is a significant interaction of CpG x Study Group p<0.05.

**Supplementary Table 4B: CpGs with Differential DNA Methylation at 28 Years and Associations with Adult Respiratory Traits, adjusting for Study Group, Sex and Ethnicity**

| **CpG and Gene** | **FEF_25–75_ z-score** | **FEV1z** | **RVz-score** | **RVz by TLC z-score** | **DLCO z-score** | **KCO z-score** | **VO_2_Max z-score** |
| --- | --- | --- | --- | --- | --- | --- | --- |
| cg13518079  ***EBF4*** |  |  |  |  | 0.012 |  |  |
| cg05857996  ***EBF4*** |  |  |  |  | 0.045 |  |  |
| cg22891070  ***HIF3A*** |  |  |  |  | 0.017 | 0.021 |  |
| cg16672562  ***HIF3A*** |  |  | 0.023 | 0.020 | 0.001 | 0.006 |  |
| cg26344859  ***KCNQ1*** | ^††^ 0.010 | ^††^ 0.034 |  | ^††^ 0.044 |  |  |  |
| cg14486095  ***UNC119B*** |  | ^††^ 0.034 |  |  |  |  |  |
| cg04099095  ***EVX1*** |  |  |  | 0.036 |  |  |  |
| cg27146050  ***HIF3A*** |  |  | 0.025 | ^††^0.032 | 0.007 | 0.029 |  |
| cg05149986  ***CFI*** |  |  |  | 0.022 | <0.001 | 0.003 | 0.040 |
| cg05825244  ***EBF4*** |  |  | ^††^ 0.048 | ^††^ 0.049 |  |  |  |
| **DNAm GrimAge Adjusted Age** |  |  |  |  | <0.001 | <0.001 | <0.001 |

Positive associations are in red, negative associations in blue. CpGs not shown in Table were not significantly associated with any trait.

^††^ Preceding the p-value indicates there is a significant interaction of CpG x Study Group p<0.05.
