# Supplementary material for: DNA methylation patterns at birth predict health outcomes in young adults born very low birthweight
Source: Clin Epigenetics. 2023 Mar 23;15:47. doi: 10.1186/s13148-023-01463-3 (PMC10035230; doi:10.1186/s13148-023-01463-3)
Supplement: Supplementary file 11 — Additional file 11: Table S5. From the lists of CpGs with differential DNA methylation in VLBW cases versus controls, four overlapped in both neonates and adults and showed several associations with adult cardiovascular and respiratory traits. [file 13148_2023_1463_MOESM11_ESM.docx]

**Supplementary Table 5: CpGs with Differential DNA Methylation between VLBW Cases and Controls in Both Neonates and Adults and Associations with Adult Traits, Adjusting for Group, Sex Ethnicity**

| **CpG**  **Gene**  Chromosome Position | | **cg16426670**  ***EBF4***  20: 2675996 | | **cg15589477**  ***EBF4***  20: 2672567 | | **cg14973857**  ***LOC101928911***  6: 88509429 | | **cg12800962**  ***SNAI1***  20: 48626437 | |
| --- | --- | --- | --- | --- | --- | --- | --- | --- | --- |
|  | | **Neonate** | **Adult** | **Neonate** | **Adult** | **Neonate** | **Adult** | **Neonate** | **Adult** |
| Methylation Direction in VLBW | | Hypo | Hyper | Hypo | Hyper | Hyper | Hyper | Hypo | Hyper |
|  | *q* value * | 1.65E^-12^ | 5.54E^-5^ | 1.63E^-11^ | 1.26E^-4^ | 1.54E^-6^ | 5.54E^-5^ | 1.47E^-6^ | 4.97E^-5^ |
|  | Fold Change | 0.029 | 1.015 | 0.919 | 1.011 | 1.052 | 1.010 | 0.960 | 1.016 |
|  | R-statistic | -0.593 | 0.241 | -0.558 | 0.221 | 0.416 | 0.236 | -0.485 | 0.251 |
| **Associations** | | | | | | | | | |
|  | Birthweight, g |  | 0.005 |  | 0.006 | <0.001** |  |  | 0.001^††^ |
|  | Gestation, wks (VLBW Cases) | 0.022 |  | <0.001 |  | <0.001 |  |  |  |
|  | BMI, kg/m^2^ |  |  |  |  |  |  |  | 0.041 |
|  | Systolic BP, mmHg | 0.037** | 0.002^††^ |  |  |  |  |  |  |
|  | LVMI_BSA, g/m^2^ | 0.017^†† ##^ |  |  |  |  |  |  |  |
|  | LVEDV_BSA, mL/m^2^ | ^††^ 0.041^##^ |  |  |  |  | ^††^ <0.001 |  |  |
|  | RV basal diam, cm |  |  | ^††^0.023^##^ |  |  |  |  |  |
|  | Endo GL Strain |  |  |  |  |  |  |  | 0.041 |
|  | Cardiac Output, L/min | 0.009 | 0.029 |  |  |  |  |  |  |
|  | Stroke Vol_BSA, mL/m^2^ | 0.030^†† ##^ |  |  |  |  | ^††^ 0.005 |  |  |
|  | Arterial Elastance | ^††^0.012 ^##^ |  |  |  |  | ^††^ 0.032 |  | 0.039 |
|  | LV Elastance |  |  |  |  | ^††^0.036 |  |  |  |
|  | Ln RHI |  | 0.013 | 0.020 |  |  |  |  |  |
|  | VO_2_ Max |  |  |  |  |  | 0.005 |  |  |
|  | FEF_25–75_ z |  |  |  | 0.017 |  |  |  |  |
|  | FEV1z |  |  |  |  |  | 0.022 | 0.047 |  |
|  | FEV1z by FVCz |  |  |  | 0.005 |  |  |  |  |
|  | RVz |  | ^††^ 0.042 | 0.023 |  |  | <0.001 |  |  |
|  | RVz by TLCz |  | ^††^ 0.045 |  |  |  | <0.001 |  |  |
|  | Prem Aging Score |  | ^††^0.039** |  |  |  |  |  | 0.042 |

* *q* value corrected for 4 CpG sites; model includes Sex, BCC Houseman (Rank), slide, position, call rate (linear).

*p* values of positive associations are in red, negative associations in blue.

^††^ Preceding the *p* value indicates there is a significant interaction of CpG x Study Group p<0.05; ^††^ following the *p* value indicates both the interaction term and CpG are significant; ** Association is significant in VLBW only; ^##^ Association is significant in Controls only.
