# Supplementary material for: DNA methylation patterns at birth predict health outcomes in young adults born very low birthweight
Source: Clin Epigenetics. 2023 Mar 23;15:47. doi: 10.1186/s13148-023-01463-3 (PMC10035230; doi:10.1186/s13148-023-01463-3)
Supplement: Supplementary file 15 — Additional file 15: Table S6. After PCA was performed on the 18 EBF4 CpGs identified from both adult and neonate methylation data, associations were observed between the first and second principal components (PC1 and PC2) at each age and adult cardiovascular or respiratory traits. [file 13148_2023_1463_MOESM15_ESM.docx]

**Supplementary Table 6: Principal Components (PC)1 and PC2 of 18 *EBF4* CpGs Differentially Methylated in Neonates and Associations with Adult Outcomes, Adjusting for Study Group, Sex and Ethnicity**

|  | | **Neonate PCA** | | **Adult PCA^1^** | |
| --- | --- | --- | --- | --- | --- |
|  | | **Association with PC1** | **Association with PC2** | **Association with PC1** | **Association with PC2** |
| **At Birth** | |  |  |  |  |
|  | Birthweight | ^††^ 0.022 | 0.025^††^ | <0.001 | <0.001 |
|  | Gestation (VLBW cases only) |  |  | 0.011 | 0.004 |
| **At 28 Years** | |  |  |  |  |
|  | Age at Screening, years | 0.029 | 0.045 |  |  |
|  | Systolic BP, mmHg | 0.029 | 0.019 |  |  |
|  | LVMI, g/m^2^ | 0.003^†† ##^ | 0.001^†† ##^ |  |  |
|  | LVEDV indexed to BSA, mL/m^2^ | 0.017^†† ##^ | 0.008^†† ##^ |  |  |
|  | LVESV indexed to BSA, mL/m^2^ |  | 0.033^†† ##^ |  |  |
|  | RV basal diameter, cm | 0.007 ^†† ##^ | <0.001^†† ##^ | 0.020 | 0.012 |
|  | RAVI, cm^3^/m^2^ |  | ^††^0.040  ^##^ |  |  |
|  | Stroke Volume indexed to BSA, mL/m^2^ | 0.006^†† ##^ | 0.011^†† ##^ |  |  |
|  | LV Elastance, mmHg/mL |  | ^††^ 0.027 ^##^ |  |  |
|  | Arterial Elastance, mmHg/mL | 0.041 ^†† ##^ | 0.038 ^†† ##^ |  | 0.031 |
|  | FEV1 z-score by FVC z-score |  | 0.044 |  |  |
|  | RV z-score |  | ^††^ 0.015 ^##^ |  | ^††^ 0.010 |
|  | RV by TLCz-score |  | ^††^ 0.036 | 0.030 | ^††^ 0.015 |
|  | DLCO z-score |  |  | 0.009 |  |

*p* values for positive associations are in red, negative associations in blue.

^††^ Preceding the p-value indicates there is a significant interaction of CpG x Study Group p<0.05; ^††^ following the p-value indicates both the interaction term and CpG is significant; ** Association is significant in VLBW only; ^##^ Association is significant in Controls only.
